# How the value of the environment controls persistence in visual search

**DOI:** 10.1371/journal.pcbi.1009662

**Published:** 2021-12-14

**Authors:** Michael R. Traner, Ethan S. Bromberg-Martin, Ilya E. Monosov

**Affiliations:** 1 Department of Biomedical Engineering, Washington University, St. Louis, Missouri, United States of America; 2 Department of Neuroscience, Washington University School of Medicine, St. Louis, Missouri, United States of America; 3 Department of Neurosurgery, Washington University, St. Louis, Missouri, United States of America; 4 Pain Center, Washington University, St. Louis, Missouri, United States of America; 5 Department of Electrical Engineering, Washington University, St. Louis, Missouri, United States of America; Dartmouth College, UNITED STATES

## Abstract

Classic foraging theory predicts that humans and animals aim to gain maximum reward per unit time. However, in standard instrumental conditioning tasks individuals adopt an apparently suboptimal strategy: they respond slowly when the expected value is low. This reward-related bias is often explained as reduced motivation in response to low rewards. Here we present evidence this behavior is associated with a complementary *increased* motivation to search the environment for alternatives. We trained monkeys to search for reward-related visual targets in environments with different values. We found that the reward-related bias scaled with environment value, was consistent with persistent searching after the target was already found, and was associated with increased exploratory gaze to objects in the environment. A novel computational model of foraging suggests that this search strategy could be adaptive in naturalistic settings where both environments and the objects within them provide partial information about hidden, uncertain rewards.

## Author summary

Classic foraging theory predicts that humans and animals aim to gain maximum reward per unit time. However, in standard instrumental conditioning tasks individuals adopt an apparently suboptimal strategy: they respond slowly when the expected value is low. This reward-related bias is often explained as reduced motivation. Here we present evidence this behavior is associated with a complementary *increased* motivation to search the environment for alternatives. We found that the reward-related bias scaled with environment value, was in part caused by persistent searching after the target was already found, and was associated with increased exploration of objects in the environment. A novel computational model of foraging suggests that this search strategy would be adaptive in naturalistic settings where both environments and the objects within them provide partial information about hidden, uncertain rewards.

## Introduction

Humans and other primates on the hunt for hidden rewards must search for them persistently, by thoroughly inspecting the objects in their environment. However, the mechanisms that motivate persistent visual search for rewards, and how they adjust behavior based on attributes of rewards like probabilities and values, are still being uncovered.

One clue comes from instrumental conditioning tasks used to study motivation. A common and seemingly straightforward paradigm is to require participants to make actions associated with different expected amounts of reward. In these settings, humans and animals consistently make actions rapidly when they are associated with large reward and slowly when they are associated with less reward or no reward [1–12]. Remarkably, they do so even when this is a seemingly suboptimal strategy that substantially reduces their reward rate. That is, because they must perform the instructed action in order to continue the task, performing slowly on trials when only a small reward is available simply prolongs the amount of time they spend in those low-value trials.

This phenomenon occurs not only for body movements to directly obtain and consume rewards, but also for eye movements as well. In oculomotor instrumental tasks, monkeys saccade to a visual target with longer latency and lower velocity when the target or its location is associated with lower reward value [5–7,13–17]. Similar behavior is found in humans as well [18–20]. These saccades often show additional hallmarks of reduced motivation, including distorted trajectories curving away from the target, and are sometimes even aimed completely away from the target (e.g. saccades toward an empty location on the screen), thus requiring multiple saccades before the target is successfully acquired [13,21].

These findings raise a critical question: how do these conserved and robust reward-related response biases observed in simple instrumental tasks contribute to an organism’s ability to search for rewards in more complex, naturalistic environments? In natural environments organisms are not presented with only one object or action at a time. Agents must persistently search for desirable objects that are hidden among many distractors in visually complex scenes [22–25]. Furthermore, organisms must adapt their foraging strategy to the richness of the environment, since behavior that is adaptive when rewards are plentiful may be maladaptive when rewards are sparse [26]. However, whether and how reward-related response biases might serve an adaptive role in such environments has not been studied in visual search.

In this study, we hypothesized that the reward-related response biases that appear to be suboptimal in simple instrumental tasks might be enhanced in more naturalistic environments where they could have an important role in regulating the persistence of visual search. To test this hypothesis, we trained two macaque monkeys to search for targets in complex visual environments. Half of the targets were stably associated with reward, while the other half were stably associated with no-reward. Each environment was associated with a different probability of containing a rewarded target.

We found that reward-related response biases were greatly enhanced in these more naturalistic environments. Furthermore, while visual search durations were indeed related to the reward value of the target, they were unexpectedly also strongly related in the *opposite* manner to the reward probability of the environment. As a result, visual search durations were longest when a low-value target was encountered in a high-value environment. Importantly, these prolonged searches were not simply due to the animals reducing their rate of taking actions or increasing their distractibility, as one might expect if they simply suffered from a general reduction of motivation. Instead, they occurred in part because animals continued to persistently search their environment, actively inspecting its constituent objects. To understand this further, we formulated a computational model of foraging that extends classic foraging theories by incorporating the need to inspect objects to learn their values. The results suggest that this search strategy could be adaptive in naturalistic situations where animals must search their environments to uncover hidden, uncertain rewards.

Taken together, this data suggests that reward-related response biases are not a simple *reduction* of motivation in the face of low-value objects, and instead may be an adaptive *shift* of motivation, away from engaging with a low-value object and toward searching the environment for alternatives.

## Results

To study the reward-related response bias in visual search, we designed a search task in which animals had the opportunity to respond at different rates depending on both the reward value of a visual targets and the reward value of the search environment in which it was embedded. Crucially, in our study, while animals could use a reward-biased response strategy, they were not encouraged or incentivized to do so. For example, just like the classic simple instrumental conditioning tasks discussed above, employing a reward-related response bias did not allow animals to improve their average reward rate.

### The reward-related response bias is enhanced during visual search

**Fig 1:**
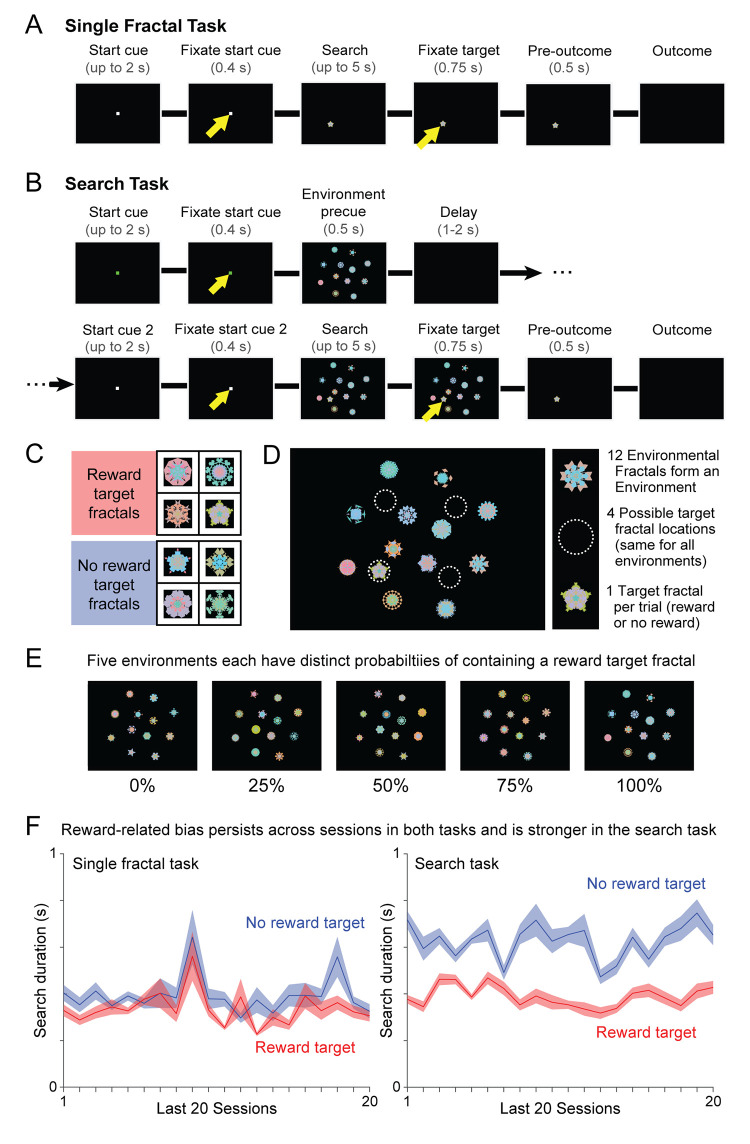
Behavioral tasks. (**A**) Single Fractal Task. Monkeys searched for a single fractal target without any surrounding environment. (**B**) Search Task. Monkeys were pre-cued with a visual environment, then searched for a single fractal target embedded in that environment. Yellow arrow indicates the required location of gaze. (**C**) One set of targets gave juice reward, while another set gave no reward. (**D**) Each environment consisted of 12 unique fractals at specific locations on screen. The single target fractal on each trial appeared randomly at one of four locations. (**E**) There were five unique environments, with different probabilities of their trial’s target being a reward target (as opposed to a no reward target). (**F**) Monkey M showed a stable reward-related bias during the last 20 sessions of both tasks, with shorter search durations on reward trials than no reward trials. This reward-related bias was stronger in the Search Task than the Single Fractal Task.

We trained two monkeys to perform a visual search task in which search targets were drawn from a pool of previously trained visual fractal objects that were associated with either reward or no reward (Fig 1A–1C). The search targets were placed in different visual environments which were associated with different probabilities of a reward target or no reward target being available (Fig 1D–1E).

We began the behavioral experiments by training monkeys to search for targets with different reward values, using a conventional instrumental conditioning experiment in which they were required to saccade to a single target presented in isolation without a surrounding visual environment (Fig 1A, Single Fractal Task). Different target fractals were stably associated with either reward or no reward (Fig 1C). The target was presented in one of four locations, and monkeys had to visually fixate it for 0.75 seconds to get the trial’s outcome (reward or no reward) and advance to the next trial. If monkeys did not fixate the target within an allotted time limit, the trial was repeated until they performed it correctly. Monkeys performed this experiment until stable within-session response time differences between rewarded and non-rewarded fractals emerged (10 sessions for Monkey M; 3 sessions for Monkey B). We will call this response time difference the reward-related response bias.

The reward-related response bias remained stable across many sessions of behavioral performance [13,25,27]. To confirm this, we embedded Single Fractal Task trials into all subsequent sessions (approximately 25% of the total trial count). Example response times for monkey M on single fractal trials are shown in Fig 1F for the last 20/107 experimental sessions. There was a clear effect of target reward value, with response times averaging 0.33 ± 0.007 seconds for reward targets and 0.39 ± 0.009 seconds for no reward targets (rank-sum test, p < 0.001). Similar results were observed in both animals (S1 Table).

We hypothesized that this reward-related bias may serve a purpose in regulating the persistence of visual search, and hence that it may be enhanced in more naturalistic search setting. To test this, we trained monkeys to search for the same targets in a visual search task (Fig 1B, Search Task). This task had a closely parallel design, with two major differences.

First, during the search phase of the task, the target was not presented in isolation. Instead, it was presented in a distinctive visual environment composed of 12 visual fractals at fixed locations on the screen (Fig 1D). The 12 environmental fractals were arranged in 3 ‘rings’ (inner, middle, and outer; Methods) each containing 4 fractals in a symmetrical arrangement. There were five possible environments, and each was stably associated with a different probability of containing a reward target (0%, 25%, 50%, 75%, or 100%; Fig 1E). The environmental fractals had not been shown to the monkeys or associated with reward before this training.

Second, monkeys were shown a ‘precue’ at the start of the trial giving them prior information about the environment that they were going to search. To do this, monkeys were required to fixate at the center of the screen, after which the upcoming environment was briefly shown for 0.5 seconds (Fig 1B, “Environment precue”). Importantly, the precue did not show them the upcoming target that they would have find, only the upcoming environment that would be the setting of their next search.

**Fig 2:**
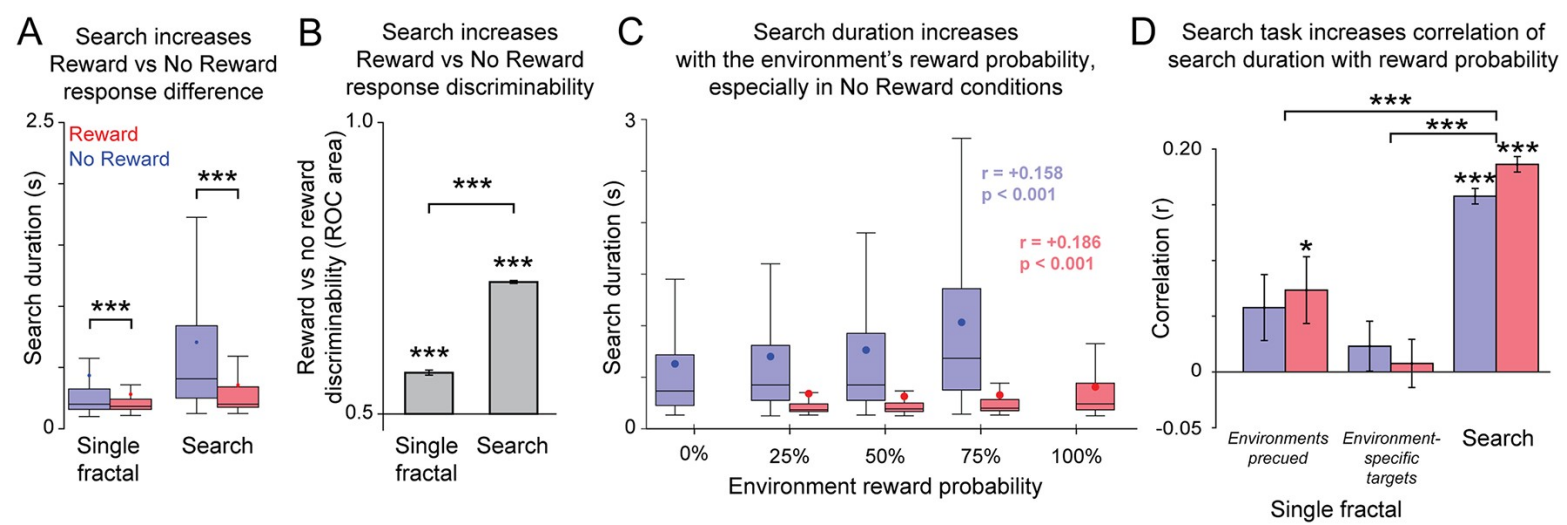
Search duration decreases with target value and increases with the environment’s probability of containing a reward target. (**A**) Distribution of search durations in each task for reward and no reward targets (red vs. blue). Box plots show medians, first and third quartiles, and limits of the data within 1.5x of the inter-quartile range, indicated by the center line, box, and whiskers, respectively. Small circles indicate the mean. *** indicates significant difference between median search durations for reward and no reward targets (p < 0.001, rank-sum test). (**B**) Mean ROC area quantifying the discriminability between search durations on reward and no reward trials. Error bars indicate bootstrapped standard error (20,000 bootstraps). *** indicates significant difference from chance (single conditions, p < 0.001, rank-sum test; comparison, 99.9% CI of the difference of Single Fractal and Search ROCs excluded zero (20,000 bootstraps)). (**C**) Distribution of search durations in the Search Task separately for each target reward value and environmental reward probability. Small circles indicate the mean. Text indicates the correlation between search duration and reward probability, and its p-value (permutation test with 10,000 permutations). (**D**) Mean correlation between search duration and reward probability, separately for the Search Task and for versions of the Single Fractal Task in which the environments were precued or for which target fractals were uniquely associated with environments (Methods). Error bars indicate bootstrap SE (20,000 bootstraps). *** indicates significance (single conditions, *, *** indicates p < 0.05, p < 0.001, permutation test with 10,000 permutations; comparisons, *** indicates the 99.9% bootstrap CI of the difference between the rho value of each of the Search conditions with the corresponding Single Fractal conditions excludes zero (20,000 bootstraps)).

Consistent with our hypothesis, the Search Task greatly increased the magnitude of the reward-related bias. This can be seen by comparing the search durations in the two tasks from the same animal in the same behavioral sessions (Fig 1F). Similar results were found in both animals (S1 Table) and are quantified in Fig 2A. The difference in search durations for reward vs. no reward targets was 0.15 ± 0.008 s in the Single Fractal Task, but grew to a much greater bias of 0.35 ± 0.006 s in the Search Task.

Importantly, the Search Task produced greater reward-related bias by enhancing the influence of reward on visual search, thus producing more distinctive patterns of behavior for reward vs. no reward targets. An alternate explanation would be that the environmental fractals simply acted as generic distractors that caused a generalized slowing of all visual searches (e.g. slowing down all searches by a fixed scaling factor). To test this, we quantified the degree to which trials with different reward values produced distinctive distributions of search durations, using the area under the receiver operating characteristic (ROC area, Methods). The ROC area was significantly above chance for both tasks, but was much greater in the Search Task (Fig 2B, 0.73, p < 0.001 for the Search Task; 0.57, p < 0.001 for the Single Fractal Task; bootstrap 99.9% CI of the difference in ROC areas is 0.140 to 0.174 and excludes zero). Thus, the reward-related bias was enhanced during visual search in a complex environment with multiple objects.

### Search duration scales in opposite manners with target reward value and environmental reward target probability

We next asked whether search duration was influenced in a similar manner by the environment’s prior probability of containing a reward target vs. no reward target (Fig 2C). We found that search durations were in fact influenced in distinct and *opposite* manners by target reward value vs. environment reward target probability. That is, while search duration was negatively related to the target’s reward value, it was strongly *positively* related to the environment’s probability of containing a reward target. Thus, search durations were short in poor environments, where reward targets were rare, and were long in rich environments, where reward targets were common.

Specifically, no reward target trials could occur in 4 distinct environments (0%, 25%, 50%, and 75%). Search duration greatly increased as a function of the environment reward probability. Searches lasted for a mean of 0.629 ± 0.008 seconds in the 0% reward environment, up to a mean of 1.03 ± 0.02 seconds in the 75% reward environment. Thus, this search duration was positively correlated with environment reward probability (Spearman’s rho = +0.158, p < 0.001). Similarly, reward target trials could occur in 4 distinct environments (25%, 50%, 75%, and 100%). These search durations increased with reward probability, lasting for a mean of 0.341 ± 0.009 seconds in the 25% reward environment, up to a mean of 0.405 ± 0.005 seconds in the 100% reward environment. Thus, this search duration was positively correlated with environment reward probability (rho = +0.186, p < 0.001). This behavior did not require the target to be at specific or expected locations on the screen. These correlations were preserved in a control experiment in which target fractal did not occur at one of its usual locations and instead replaced one of the environmental fractals (rho = +0.213 and +0.203 for no reward and reward respectively, both p < 0.001; Methods). Thus, searches were prolonged in environments where reward targets were common, and this occurred consistently regardless of whether the target on screen during the current trial was the reward target or the no reward target. The same same pattern was observed for peak saccade velocity as search duration for Monkey M (Reward: rho = +0.13, p < 0.001 No Reward rho = +0.02, p = 0.03) and Monkey B (Reward: rho = +0.11, No Reward rho = +0.08, both p < 0.001).

Furthermore, the correlation between search duration and reward probability was significantly stronger during the Search Task that required visual search in a complex environment, than during the Single Fractal Task which did not (Fig 2D). This was true regardless of how the trial’s reward probability was indicated to the animal. Notably, this result occurred even in a version of the Single Fractal Task in which the environmental reward probability was explicitly cued to the animal on each trial, using the same environmental precue as the Search task (Fig 2D, “Environments precued”; Methods). This result also occurred in a version of the task in which the environmental reward probability was indicated by a fixed association between each individual target fractal and a specific environmental reward probability (Fig 2D, “Environment-specific targets”; Methods). For example, in the Search Task one specific reward target fractal was only presented in the 25% reward environment; another was only presented in the 50% reward environment; etc. When those same target fractals were later presented in the Single Fractal Task, however, the search duration had little correlation with their associated environment’s reward probabilities (Fig 2D, environment-specific targets, rho = +0.023, p = 0.296 for no reward, rho = +0.008, p = 0.751 for reward; environments precued, rho = +0.058, p = 0.053 for no reward, rho = +0.073, and p = 0.014 for reward; all are lower than the corresponding correlations in the Search Task, indicated by the 99.9% bootstrap CIs of the difference between correlations excluding 0).

Thus, search duration scaled in opposite manners with target reward value and environmental reward probability, and these effects were much stronger during search for a target in a complex visual environment.

### The reward-related bias was not incentivized by the task

A key point is that animals developed and maintained a strong reward-related bias in the Search Task even though the task did not incentivize them to do so. That is, having a reward-related bias did not allow animals to increase their reward rate. If anything, having a reward-related bias appreciably *reduced* the animal’s reward rate. This is consistent with previous research using simple instrumental conditioning tasks, in which animals often get a lower reward rate in tasks that permit a reward bias (e.g. when a cue indicates whether each trial will provide reward vs no reward; [13,28,29]). This results from animals spending more time on trials that they know will be non-rewarded, due to making slower responses and committing more errors.

To check if this was the case in our task, we calculated the ratio between the actual reward rate that animals achieved from the search period, and the theoretical reward rate they *would* have achieved if they had behaved in the same manner on non-rewarded trials as rewarded trials (Methods, S1 Fig). This reward rate ratio accounted for the time animals spent to complete search trials correctly and the time they spent on search errors when they failed to fixate the target within the allotted maximum search time. This produced three main findings (S1 Fig). First, the actual reward rates for all animals were considerably lower than what they would have achieved if they had performed with constant motivation (all reward rate ratios < 1 with 99.9% CIs excluding 1). Second, this deficit in reward was significantly greater in the Search Task than the Single Fractal Task (0.84 vs. 0.86 in animal B; 0.91 vs. 0.97 in animal M; differences between the ratios had 99.9% CIs excluding 0). Third, this deficit in reward was even greater on the subset of trials where reward-biased strategies would be expected to have the greatest effect–on trials when the animal knew that both reward and no reward targets were possible, and hence could have an accentuated behavioral bias between them (S1 Fig). Thus, while the Search Task enhanced the reward-related behavioral bias (Fig 2), this behavioral strategy was not incentivized by the task, and if anything produced a *reduction* in the actual reward rate.

### Search duration effects are robust to controlling for sensory and motor features of visual search

What mechanism is responsible for prolonging visual search when the target’s value is low and when the environment’s value is high? We hypothesized that this behavior is part of the monkey’s strategy for searching for rewards: that the monkey is fully capable of perceiving and moving to the target, but chooses not to hold fixation on the target and immediately accept the outcome, and instead chooses to continue exploring the rich high-value environment.

However, there were at least three alternative explanations for the effects of environmental reward probability. (A) Perceptual: the monkey is less able to perceive the target. (B) Motor vigor: the monkey is able to perceive the target but less willing to expend motor effort (e.g. in order to conserve energy), resulting in a reduced saccade rate and slower or less accurate responses. (C) Focus on the task: the monkey is fully capable of perceiving and moving to the target, but is more easily distracted by non-task-related stimuli, and hence takes longer to focus on the task stimuli and fixate on the target.

**Fig 3:**
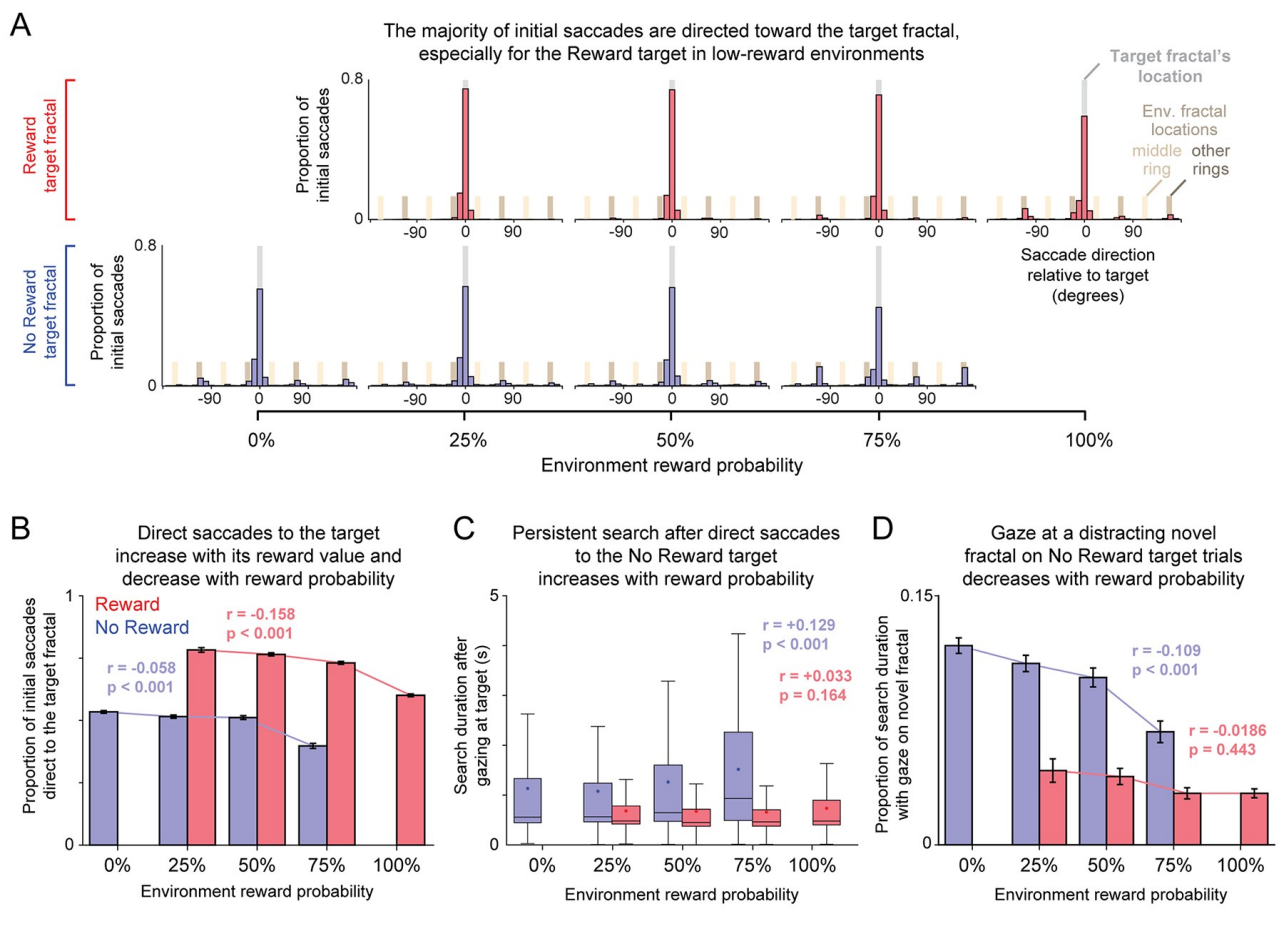
Search effects are robust to perceptual and motor features of visual search. (**A**) Histogram of the initial saccade’s angular direction relative to the location of the target fractal, separately for each target reward value (rows) and environmental reward probability (columns). Gray, light tan, and dark tan shaded areas indicate the radial angle locations of the target fractal, middle ring of environmental fractals, and other rings of environmental fractals (inner and outer rings). (**B**) Proportion of initial saccades that were direct saccades to the target fractal (landing within 1.5° visual angle of the target). For each target reward value, text indicates correlation between the proportion of direct saccades and reward probability, and its p-value (permutation test, 10,000 permutations). Error bars indicate SE. (**C**) Remaining search duration after animals made an initial direct saccade to the target and then shifted their gaze away from it. Same format as Fig 2C. (**D**) Proportion of search duration spent gazing at an unexpected trial-unique novel fractal (gaze within 1.5° visual angle) in a control task in which such novel fractals replaced a random environmental fractal. Same format as Fig 3B. Error bars indicate SE.

To distinguish between these mechanisms, we first examined whether the monkey’s initial saccade was in the direction of the target. These provide evidence on whether monkeys were able to perceive the target quickly enough to influence their initial saccade. Indeed, we found that the majority of initial saccades (79.8%) were angled within 15° of the target. This was the case for all environments and target values (Fig 3A). On a smaller proportion of trials, the initial saccade was directed elsewhere, generally in the direction of one of the environmental fractals that were nearest to the central fixation point. This occurred most often on trials when the target value was low and the reward probability was high (Fig 3A). This data indicates that the hypothetical perceptual and motor vigor mechanisms could have influenced behavior on this fraction of trials when the monkey’s initial saccade was away from the target.

However, what about the many trials when the monkey’s initial saccade was directed at the target? To examine this, we selected the subset of trials in which the monkey’s initial saccade landed at a location within 1.5° of the center of the target, which we will refer to as “direct saccades". As predicted, these direct saccades occurred most often when the target value was high and the environmental reward probability was low (Fig 3B).

We next asked whether these direct saccade trials showed the modulations of search duration that we observed in the average behavior. Any variation in search duration on these trials would be remarkable because animals had already found the target–displaying behavior consistent with perceiving the target and moving vigorously enough to hit it with an immediate direct saccade. Thus, any remaining variations in search duration are less likely to reflect perceptual or motor limitations and may instead reflect the search strategy employed by the animal.

We found that there were indeed large variations in search behavior even after a direct saccade, which were strongly influenced by both target value and the environmental reward probability (Fig 3C). Specifically, when monkeys made a direct saccade to a no reward target, being in an environment with high reward probability made them far more likely to break fixation on the target and continue their search (61.6 ± 1.6% vs. 30.4 ± 0.7% of the time in the 75% reward vs. 0% reward environments), to break fixation with a shorter latency saccade away from the target (228.4 ± 5.4 ms vs. 321.9 ± 5.0 ms), and to carry out the remaining portion of their search for a longer duration (1.52 ± 0.06 s vs. 1.13 ± 0.03 s; quantified as the time period between the animal’s gaze leaving the target and the end of the search period, either by successful fixation on the target or by elapsing the maximum search duration). Thus, the remaining search duration was significantly and positively correlated with reward probability (Fig 3C, rho = +0.129, p < 0.001; this occurred consistently in both animals (S1 Table)). We did not observe such clear modulations by environmental reward probability when monkeys made a direct saccade to a reward target, perhaps due to a floor effect, but did observe clear differences in behavior between direct saccades to reward targets vs. no reward targets. Monkeys had a uniformly low probability of breaking fixation to continue search after directly saccading to a reward target (17.6 ± 0.3% of the time) and carried out such further searches for a short remaining duration (0.69 ± 0.01s, Fig 3C). This indicates that on trials when monkeys were little affected by perceptual and motor limitations, both target value and environmental reward probability still influenced the monkey’s search strategy.

Finally, could search durations be modulated by target value and environmental reward probability by simply altering the animal’s general level of focus or engagement with the task? For example, perhaps being in a rich environment causes animals to enter a state of high excitement and distractibility, where they have normal perception and vigor of motor actions but cannot settle down and hold their gaze on task-related stimuli, and instead are prone to gaze at random salient objects or locations unrelated to the task.

To test this, we did a control experiment in one animal in which, on half of trials, a random member of the middle ring of environmental fractals was replaced by a randomly generated, trial-unique novel fractal (Methods). Novel objects are highly salient to monkeys and can potently attract their gaze [30–33], including the specific type of novel fractal objects we used in this experiment [34]. Therefore, if high reward environments induce a state of heightened distractibility, they should cause monkeys to spend a greater fraction of their search time gazing at the unexpected novel object.

Instead, exactly the opposite was the case (Fig 3D). When the environment had high reward probability, the animal spent a *smaller* fraction of its search duration gazing at the novel fractal on no reward target trials (rho = -0.109, p < 0.001), and did not display a significant trend for reward target trials (rho = -0.0186, p = 0.454). Furthermore, gaze at the novel fractal was also strongly affected by target reward value, attracting gaze for a much smaller fraction of search duration when the search array contained a reward target (Fig 3D). Thus, if anything, rich environments and high value targets caused an *enhanced* focus on the task and its stimuli.

### Enhanced reward-related response bias occurs due to persistent exploration of objects in the environment

Our data thus far suggests that rich environments caused animals to become more focused on the task. Yet in those same environments animals took a much longer time to successfully fix their gaze on the target and complete the trial, even after they had already found the target. What were they doing during these prolonged visual searches to produce the reward-related bias?

**Fig 4:**
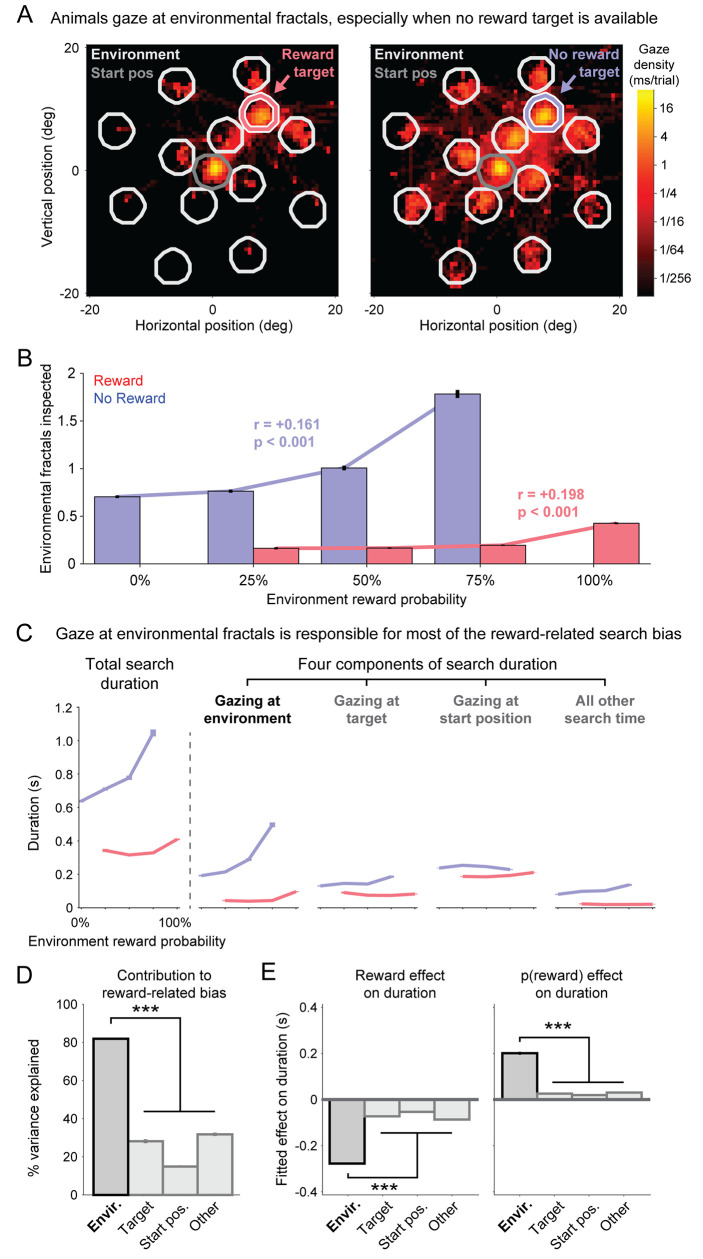
Prolonged search durations are spent actively inspecting objects in the environment. (**A**) Heatmaps of gaze distributions from animal B during the Search Task in the 50% reward environment, on trials with a reward target (left) or no reward target (right) located at the upper right target location. Areas bordered in white, colored, and gray lines indicate the analysis windows for gaze on the environmental fractals, target, and central starting position. (**B**) Number of environmental fractals inspected separately for each target reward value (red vs blue) and environmental reward probability. Same format as Fig 3B. (**C**) Mean total search duration for each target reward value and environmental reward probability (left), and the mean duration of each of the four components of the search duration. Error bars indicate SE. (**D**) Percent of reward-related variance of the total search duration that is explained by subtracting each of the four components of the total search duration. Error bars indicate bootstrap SE. *** indicates that the 99.9% bootstrap CIs of the difference between the environmental component vs. each other component all exclude 0. (**E**) Main effects of target reward value (left) and environmental reward probability (right) on each component of the total search duration, fitted by ordinary linear regression. Same format as D.

We found that animals continued to actively search their visual environment, with an especially high concentration of their gaze on the environmental fractals (Fig 4A). That is, in addition to spending time at the starting position at the center of the screen (Fig 4A, gray circle) and gazing at the target (Fig 4A, colored circles), animals also spent considerable time gazing at the 12 environmental fractals (Fig 4A, white circles). Animals gazed particularly often at the environmental fractals in the vicinity of the target, but there was some gaze allocation to distant environmental fractals as well, even those in the opposite direction of the target. Importantly, animals did this even though gazing at the environmental fractals had never been rewarded. In fact, gazing at environmental fractals had only ever caused future rewards to be delayed, by prolonging the search duration before the target could be fixated and the trial could be successfully completed.

Particularly striking was the fact that animals responded to the presence of a no reward target by greatly increasing their gaze at environmental fractals. The no reward target provided negative feedback to the animal, indicating that no reward would be available during the trial. One might imagine that animals would be demotivated by this negative feedback–perhaps they would ‘space out’ by looking away from the environment, dwell on the space between the fractals, or ‘reduce action’ by reducing their number of saccades. Instead, monkeys displayed exactly the opposite behavioral strategy: they made saccades to many more environmental fractals, actively exploring more of the objects in their environment (Fig 4A).

Indeed, while we had observed reward-related biases in visual search duration as a whole, those biases were particularly strong during the component of search when the animal was gazing at environmental fractals. When a reward target was available, animals allocated relatively little search time to gazing at environmental fractals, and typically did so in the immediate vicinity of the target (Fig 4A, left). However, when no reward target was available, animals allocated much greater search time to gazing at environmental fractals, even ones distant from the target (Fig 4A, right). Thus, animals inspected more environmental fractals when the target value was low (Fig 4B, no reward > reward; p < 0.001, rank-sum test). In addition, animals inspected more environmental fractals when the environment’s reward probability was high (Fig 4B), doing so during both reward target trials and no reward target trials (Fig 4B, red and blue bars; reward trials r = +0.198, p < 0.001; no reward trials r = +0.161, p < 0.001).

To measure how much of the reward-related search bias was due to gaze at environmental fractals, we measured how reward probability and target reward value influenced the total search duration (Fig 4C, column 1), as well as how they influenced four separate components of the total search duration: gaze at environmental fractals, gaze at the target, gaze at the central starting position, and all remaining search time (Fig 4C, columns 2–5). There were clear and consistent reward-related biases in all four components of visual search. However, the effects of reward probability and target reward value were by far the largest in the component of search spent gazing at the environmental fractals (Fig 4C, column 2). This suggests that gaze at environmental fractals was the dominant contributor to the reward-related bias in visual search. Also this could suggest a reason for the widely-observed phenomena that response times to unrewarded targets are slower, even in Single Fractal / object tasks (where the value of the environment is simply the mean reward rate).

To quantify this, we measured the amount of reward-related search duration variance, by calculating the variance of total search duration across the 8 reward-related conditions (consisting of the 8 possible combinations of target reward value and environmental reward probability). We then calculated the percentage of this reward-related variance that could be explained by each of the 4 separate components of visual search (Fig 4D). Note that the percentages of variance explained sum to greater than 100% because the animals allocated time to the four components of search in a partially correlated manner. For example, on trials when the animal gazed at many environmental fractals, they necessarily also spent more time in transit saccading between them. We therefore tested which of the four components of search best explained the reward-related variance in total search duration.

We found that gaze at the environmental fractals explained by far the greatest percentage of reward-related variance in search duration, explaining significantly more than each of the other three components of search (Fig 4D). As a second test, we fit each component of search duration with a simple linear model including the two reward-related factors. All four components of search were best fit by a significant negative effect of target value and a significant positive effect of reward probability, but both of these effects were significantly greater for gaze at the environmental fractals than for any other component of the total search duration (Fig 4E).

Importantly, the predominant contribution of environmental gaze to the reward-related bias was not simply due to environmental gaze having a longer duration or greater variability than the other components of search. This could be seen by the fact that environmental gaze was similarly and significantly predominant in both animals, despite the two animals allocating mean search duration and variability quite differently to the four components of search (S2 Fig). Specifically, animal B’s environmental gaze had a significantly higher mean and variability than all other components of search, while animal M’s environmental gaze had significantly *lower* mean and variability than other components of search (S2 Fig). This indicates that the reward-related bias in visual search is not caused by a simple, generalized change in motivation that affects all components of search equally. Instead, the reward-related bias was particularly attuned to opportunities to gaze at the environment and the objects that compose it.

### An extended model of foraging incorporates the need to inspect objects in the environment to find hidden, uncertain rewards

Given our findings on the nature of reward-related bias during visual search, we next asked what purpose this behavioral strategy could serve. Animals persistently continued using this strategy despite extensive training, even though it did not maximize reward rate in our task. This suggested that animals are not using this strategy because it is optimized for our specific task, and instead because it is adaptive in alternate environments, such as the ones they would encounter while foraging in nature.

In particular, our task and related instrumental conditioning tasks are purposefully designed to perform a controlled dissociation of the effects of targets and distractors. Each environment contained one object that could potentially yield reward and would allow the animal to advance to the next trial (the “target”), with a fixed reward probability and magnitude. All of the other objects always yielded zero reward (the “distractors”). In many natural environments, however, there is no categorical separation between “target” and “distractor.” Foraging environments contain multiple objects that could potentially yield reward, with a continuum of different reward probabilities and magnitudes.

We hypothesized that the reward-related bias we observed could be beneficial in such natural environments by helping animals navigate the tradeoff between exploration and exploitation. Discovering that a target is valuable should encourage animals to respond to it to obtain its outcome (i.e. negative effect of target value on response time). However, being in a rich environment should encourage further exploration before collecting the reward, due to the chance to find even more value from additional targets (i.e. positive effect of environmental value on response time). Therefore, an agent that optimized its foraging strategy for such naturalistic scenarios might scale its response times with target and environment values similarly to the monkey behavior we observed.

**Fig 5:**
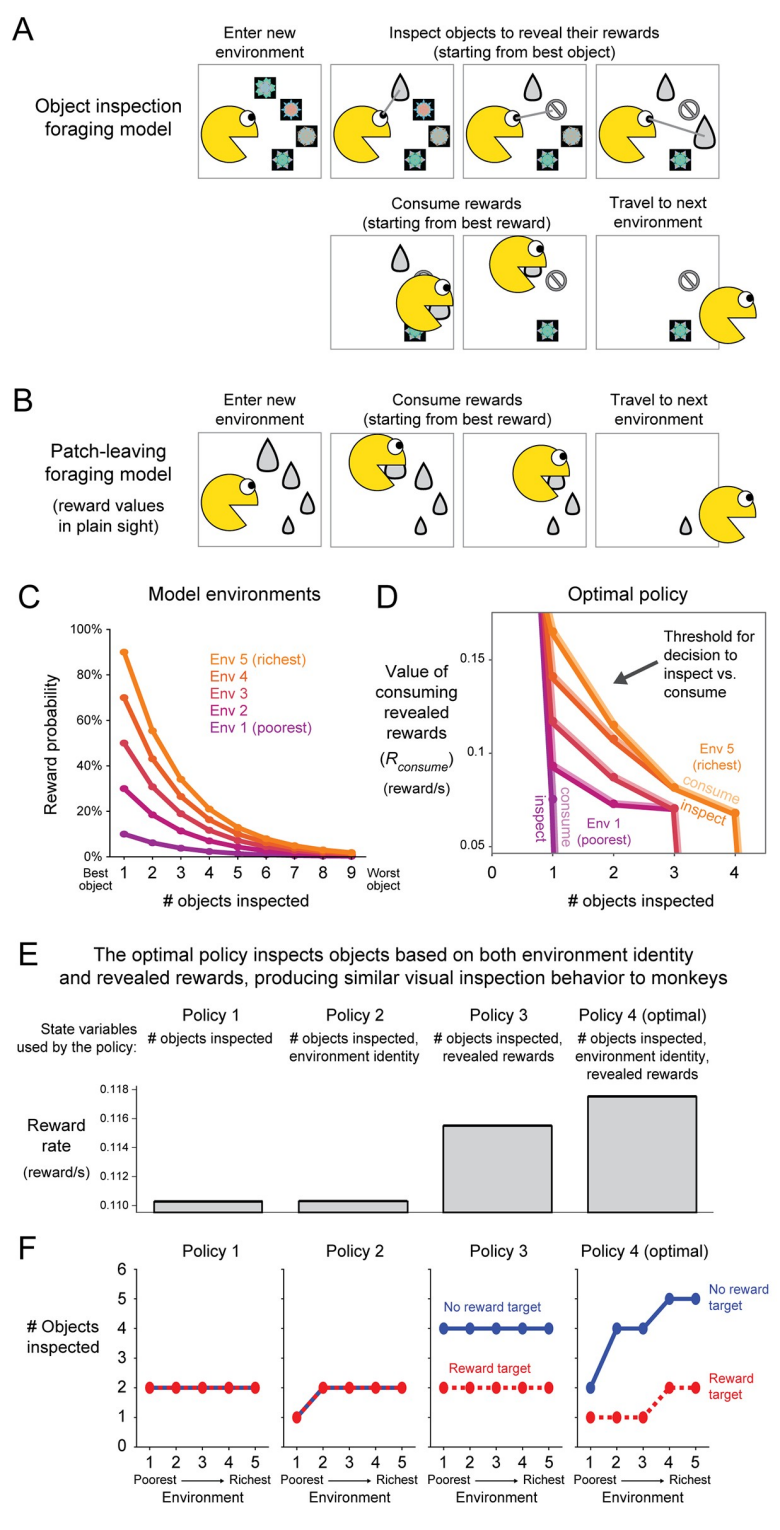
Persistent visual search in rewarding environments is adaptive in a foraging model where inspecting objects is required to reveal uncertain rewards. **(A)** Object inspection foraging model. The agent enters a new environment, inspects a subset of objects to reveal rewards (starting from the best object), consumes a subset of rewards (starting from the best reward), then travels to a new environment. **(B)** Classic patch-leaving foraging model. All rewards are directly perceived without any requirement for object inspection, so there is no object inspection phase, only a consumption phase. **(C)** Object inspection model of rich vs poor environments. Each environment has 9 objects that yield reward with different probabilities. Richer environments have higher reward probabilities. Colored lines indicate the reward probability of each object in each of the 5 environments. **(D)** Optimal policy for object inspection. Each location on the 2D plot corresponds to specific properties of the foraging model’s state: the x-axis indicates the number of objects inspected so far, the y-axis indicates the local reward rate that would be obtained by immediately starting the consumption phase to consume the rewards that have been revealed thus far. The colored line for each environment indicates the policy’s decision boundary for choosing to continue inspecting more objects (points below the line) vs. choosing end the inspection phase and begin the consumption phase (points above the line). The lines are higher in rich environments, indicating that the policy has a bias to inspect more objects in rich environments. (**E**) Reward rate achieved by optimized policies that have access to different features of the state (indicated by text). Performance is low for policies that do not have access to the revealed rewards (1, 2), is higher for a policy that has access to revealed rewards (3), and is highest for a policy that has access to the full state including both the revealed rewards and the environment’s identity (4, “optimal policy”). (**F**) Behavior of the policies when applied to a simulation of our visual search task, in which the first ‘target’ object reveals reward (red) or no reward (blue) and the remaining objects reveal no reward. Policies 1–3 behave very differently from monkeys, while the optimal policy 4 behaves similarly to monkeys: inspecting more objects when target value was low (blue > red) and environmental reward probability was high (environment 5 > 1).

To test this hypothesis, we formulated a computational model of foraging in environments with multiple objects and uncertain rewards (Fig 5A and 5B). We started with a version of the classic patch-leaving task that is fundamental in foraging theory [26,35] and has been used in neuroscience to study the neural circuits underlying foraging in humans and animals [36–41] (Fig 5B). The model world consists of multiple environments (called “patches”) that have different reward rates (e.g. rich vs. poor). An agent initially enters a patch and begins to consume its rewards. We follow the standard practice of modeling the reward rate as decreasing with time in the patch (“patch depression” [26]), which we model here as the result of the agent starting with the best rewards in the patch before moving on to progressively less valuable rewards (Fig 5B). Consuming each reward comes at a time cost (which we will call *t_consume_*). At any time the agent is free to leave the patch, incurring a travel time cost (*t_travel_*) and then arriving at a random new patch. Thus, the agent’s key decision is how long to forage in the current patch before leaving to travel to a new patch. This is similar in many ways to our task, which has different environments with different reward rates, and in which the agent’s key decision is when to finish foraging in the current environment (i.e. make their response) and then incur a travel time cost (i.e. the inter-trial interval) to enter a new random environment.

The classic approach to modeling behavior in this task is to derive the optimal patch leaving time using the marginal value theorem [35]. However, this does not account for the ability of animals to visually search the objects in their environment to learn about their reward values. Specifically, the theorem requires the agent to have perfect knowledge of the patch’s *gain function g_i_(t_i_)* which fully describes how the reward rate will decrease over time as the agent harvests its rewards [35]. In effect, all of the rewards are in plain sight, and the agent immediately knows their values without requiring any further inspection (Fig 5B). This is an immense advantage–akin to playing poker with the cards face-up. In natural environments, of course, this knowledge often comes at a cost [26]. Animals must spend time and effort carefully surveying the objects in their environment to learn which rewards are available and where they are located.

We therefore extended the classic model by adding a requirement to visually inspect objects in order to discover their hidden, uncertain reward outcomes (“Object inspection foraging model”, Fig 5A). In this model, each time the agent enters a new patch, they are faced with an array of objects that deliver probabilistic rewards. The agent makes a rapid initial assessment of each object’s expected reward value, analogous to the first-pass visual information an animal obtains about an environment before making saccades to inspect its objects. In rich patches many objects will have high expected values, while in poor patches most objects will have low expected values (Fig 5C). We modeled this by setting the object reward probabilities to be an exponentially decaying function of object number, starting at 90% reward for the best object in the richest patch and starting at 10% reward for the best object in the poorest patch (Fig 5C). Other ways of modeling rich vs poor patches gave qualitatively similar results (S3 Fig).

The agent then forages in two phases. In the first phase, the *inspection phase*, the agent inspects objects in order to reveal their outcomes, starting by inspecting the best object (the one with the highest expected value) and moving on to objects with progressively lower expected values. Inspecting each object comes at a time cost (*t_inspect_*). This is roughly analogous to the animals in our experiments spending time to attend and saccade to the target stimulus in order to find out whether it will provide a reward or no reward. The agent may choose to stop the inspection phase at any time. The agent then moves on to the second phase, the *consumption phase*. This is the same as the classic foraging model: the agent consumes the revealed rewards one by one in order of their values, until they choose to stop consumption and travel to a random new patch. As in natural environments, the agent must inspect an object before revealing and consuming its reward. Finally, because our model includes situations where sensory cues (objects) indicate delayed rewards, it must account for the fact that delaying rewards in natural environments can reduce their value [42]; for example, a predator that dallies before the hunt may lose sight of its prey. To model this, we include a temporal discounting rate (*k*) to control how reward values decay with elapsed time (Methods). This incentivizes the agent to balance its time inspecting objects vs. consuming their rewards.

In summary, the object-inspection foraging model extends the classic patch-leaving task by requiring objects to be inspected to reveal their outcomes, and introducing uncertainty due to outcomes being probabilistic.

### Reward-related response bias modulation by environment value is optimal when both environment identity and visual objects provide cues to find hidden rewards

In this setting, what is the optimal strategy for inspecting objects in order to maximize the reward rate? The agent must compute the reward rate that would result from continuing to inspect more objects (*R_inspect_*) and compare it against the reward rate that would result from ending the inspection phase and consuming the revealed rewards (*R_consume_*).

We calculated the optimal policy using dynamic programming (Fig 5D) and found that its strategy could be interpreted as follows. The optimal policy computes *R_inspect_* using two variables: the richness of the current patch, and the number of its objects that have been inspected. These allow the agent to estimate the expected reward value of the next best object that has not yet been inspected, and thus, to estimate whether it is worth expending *t_inspect_* to inspect that object and reveal its outcome. The optimal policy computes *R_consume_* using the marginal value theorem, by using the gain function implied by the current set of revealed rewards to decide how many of them are worth expending *t_consume_* to consume before leaving to go to a new patch.

The optimal policy balancing these two values shows both of the key features we observed in monkey object-inspection behavior (Fig 5D). First, inspection times were strongly negatively related to the amount of obtainable reward the agent had revealed thus far, as quantified by *R_consume_*. That is, for any fixed environment and number of inspected objects, the optimal policy set a decision threshold based on *R_consume_* (Fig 5D, dots). If *R_consume_* was below the threshold the agent decided to continue inspecting in hopes of finding more rewards, while if *R_consume_* was above the threshold the agent decided to finish inspecting and consume.

Second, and crucially, inspection times were strongly *positively* related to the environment’s richness (Fig 5D). This makes intuitive sense. After inspecting the best object in an environment, a rich environment is still likely to contain more valuable rewards, while a poor environment is not likely to contain further rewards worth collecting. Thus, the decision threshold was positively related to environmental richness (Fig 5D). In rich environments the decision threshold was shifted upward; the optimal agent continued to inspect objects unless the total revealed rewards were very high, or many objects had been inspected (Fig 5D, orange). In poor environments the decision threshold was shifted downward; the optimal agent only inspected more objects if the currently revealed rewards were very low, and even then, it only continued the inspection for one or two more objects at most (Fig 5D, purple).

All of these variables were necessary for the optimal policy to achieve its high reward rate (Fig 5E). In fact, they had a synergistic effect. Agents that only had access to a subset of these variables could only achieve relatively lower reward rates, even though their policies were optimized to make the best possible use of that limited information (Fig 5E, left). An agent that had access to all of these variables, and thus used the overall optimal policy (Fig 5D), achieved a noticeably higher reward rate (Fig 5E, right). Thus, optimal visual search in this setting requires accurate knowledge of both the currently visible rewards and the richness of the environment.

Finally, we asked whether the optimal policy for this naturalistic object-inspection foraging task would behave similarly to our monkeys, if it was applied to a visual search task like the one the monkeys performed (Fig 5F). To test this, we examined the policy’s behavior during trials in which the first, best object that it inspected (analogous to the “target” object in our visual search task) revealed either an average size reward or no reward (“rewarded target” or “non-rewarded target”). Moreover, we specifically examined trials in which the remaining objects that it inspected all revealed no reward (analogous to the remaining “distractor” objects in our visual search task).

Indeed, the optimal policy behaved similarly to the monkeys (Fig 5F, right). The agent inspected more objects when the target was non-rewarded than when it was rewarded (blue > red), and inspected more objects in rich environments than poor environments (both blue and red lines have slopes > 0). This result was not sensitive to the specific details of the model. Qualitatively similar results occurred for many different ways that naturalistic environments can be rich vs poor, including (1) scaling up the reward probabilities of all objects, (2) increasing the number of objects with high reward probabilities, (3) scaling up the reward magnitudes of all objects, (4) all of the above combined (S3 Fig). Furthermore, this behavior during visual search only occurred for agents that were using the optimal policy for the object inspection foraging task. It did not occur for any of the agents that only had access to a subset of the necessary state variables (Fig 5F). For example, agents lacking access to the revealed rewards gained much less benefit from inspection and hence never inspected more than two objects (Fig 5F, Policies 1 and 2), while agents lacking access to the environment identity had to resort to identical behavior in all environments (Fig 5F, Policy 3). Finally, as in monkey behavior, the optimal policy’s number of inspected objects was more influenced by its prior knowledge of the environment’s richness in situations where no rewards had been found thus far, compared to situations where one reward had already been found (blue slope > red slope, Fig 5F; this occurred consistently in all of the alternate versions of the model described above, S3A–S3D Fig). Indeed, this pattern of behavior was strongly and significantly present in each individual animal (blue slope > red slope, Fig 4B for pooled data, S3 Fig for individual animals; bootstrap 99.9% CI of difference between best-fit linear regression slopes excluded 0 in both pooled data and in each animal).

This suggests that the ‘suboptimal’ monkey behavior during our task may have resulted from monkeys using a strategy that is optimized for a more naturalistic setting. Specifically, their behavior would be adaptive in a setting where inspecting the best object in view and revealing a bad outcome is not simply a cause for despair, but is also a spur to action to continue inspecting more objects to find better outcomes, especially in environments that are known to be rich.

## Discussion

We found that the reward-related bias seen in simple instrumental tasks was strongly enhanced when monkeys had to find targets in a more naturalistic multi-object visual search environment. Monkeys had two strong biases during visual search: they prolonged their search when the target was low value and when it was encountered in a high-value environment. They implemented these biases by changing their search strategy: they shifted their focus away from gazing at the target, and instead allocated additional time to persistently inspecting the other objects in the environment. Monkeys continued to use this strategy over the course of extensive training, even though it was suboptimal in our task and substantially reduced their reward rate. We found that monkeys’ strategy in our task resembles the optimal strategy to gather rewards in a more naturalistic foraging scenario, if one extends a classic model of foraging to incorporate the requirement that objects must be inspected to discover their hidden and variable reward values.

Our findings shed light on the potential purpose of a common, robust, and highly conserved pattern of behavior: animals make slower responses to low-value objects than high-value objects. We found that monkeys employed this behavior as one component of a broader strategy during visual search, such that they regulated the persistence of search based on both target reward value and environmental reward probability. This strategy had the net effect of shifting their focus of gaze away from low-valued objects and toward exploring high-value environments–a strategy that would be adaptive in many natural environments.

In support of this view, the reward-related bias was strongly influenced by the nature of the task environment. The bias was present in a conventional instrumental conditioning task in which only a single, target object was visible (replicating previous findings with similar stimuli [27,43,44]). But importantly, the bias was expressed much more strongly in a more naturalistic search task where the target was embedded in a visual environment with multiple objects that could be explored. Indeed, the major reason the animals’ strategy resulted in slowed responses to low-value targets was because they allocated considerable time–often multiple seconds–to exploring the environmental objects even after finding the target, especially in environments associated with a high probability of reward. These results support and extend previous work showing that primate gaze is not only attracted to high-value objects but can actually be repulsed by low-value targets [14,44,45]. Notably, our results suggest that the low reward response time bias does not involve a purely negative form of motivation, such as a simple repulsion of gaze from low-value objects or their locations. Instead, in naturalistic environments, such as visual search, the same mechanism also spurs a positive form of motivation–causing gaze to be attracted to other objects in the environment, effectively igniting a search for alternative sources of reward.

Our task design and controls addressed several alternative explanations for the reward-related bias in our tasks. (1) Animals may be less able to perceive and respond to undesirable low-value targets than desirable high-value targets [14,46–48]. However, the bias persisted even after animals selected the target with an immediate, direct saccade and placed it at the center of gaze. (2) Animals can reduce their vigor in low-value situations to conserve energy [49–52]. However, if anything, animals did the reverse in our task. After fixating a low-value target, they had the option to immediately escape the low-value situation by simply holding their gaze on the target for a brief time. Instead, they often chose to shift their gaze away and make a series of saccades to inspect multiple objects in the environment. (3) Animals may cope with low-value trials by reducing their level of engagement with the task as a whole, or their focus on task-relevant objects [8,53]. However, under the conditions when the bias was strongest, animals were *more* focused on task-related objects, shown by their greater resistance to unexpected, novel distractors.

Our approach has several limitations. First, most of our key analyses and results concentrate on the search trials, and our computational model is also entirely based on visual search. Therefore, it is possible that these results do not fully generalize to single object trials. If so, these results may not explain the cause of the reward-related biases and behaviors that occur in situations with a single target. Second, our approach cannot exclude or distinguish the contribution of “learned” attentional strategies or biases and the neural circuits that support them. We discuss this in the subsequent section. In particular, value driven salience may explain how the interaction between objects and the environment produces response time biases in our task and other visual search tasks [54–57]. Relatedly, we do not dissociate whether actions are made as a result of goal-directed planning or automatic, habitual behavior. If foraging is conceived of as a series of goal-directed, planned actions to maximize reward, this could limit the applicability of our model to explain the experimental data. This is because even if animals followed the model’s foraging strategy in naturalistic environments where it is adaptive for maximizing reward, they would be expected to quickly discard this or any other foraging strategy in environments where it is maladaptive for maximizing reward, such as the experimental tasks here. We hypothesize that the reward-related bias could be due to many factors, and next discuss how neural circuits could support it in the service of adaptive behavior.

What neural circuit and behavioral mechanisms could contribute to this reward-related bias in visual search? While our study does not allow us to specify a precise mechanism, it does pinpoint several key ingredients that are necessary to generate this behavior: the brain must decide when to prolong visual search, which objects to inspect while doing so, and which situations warrant the greatest degree of inspection. Interestingly, all three of these ingredients could be provided by motivational processes implemented by closely related basal ganglia circuits, and furthermore, may underlie strategy switching in visual search foraging behavior.

First, our data indicates that the reward-related search bias occurred in situations that evoked strong “reward prediction errors” (RPEs). RPEs are signaled by key neural populations, including lateral habenula and midbrain dopamine neurons, that regulate motivation and instruct reinforcement learning in cortex and basal ganglia circuits [58–60]. RPE is roughly defined as “actual reward–predicted reward”, and likewise, search durations in our task were speeded by actual reward (target value) and lengthened by predicted reward (environmental value). This extends previous reports that neural RPEs and saccade biases change in parallel during learning [61–64] and RPEs influence saccade vigor in humans [65]. In particular, our data indicates that events that trigger negative RPEs (“less reward than predicted”) do not necessarily cause a global reduction in motivated behavior, and can instead shift motivation away from engaging with the responsible object and toward exploring alternative parts of the environment. Also, these processes likely contribute to object-salience learning, such as through value driven salience mechanisms [55], and may influence relatively more automatic behaviors, not just goal directed ones based on online evaluations of value.

Second, our data indicates that in these situations, gaze was directed toward objects based on their long-term statistical associations with reward. Notably, gaze at environmental objects was enhanced by their long-term association with high reward rates (rich environments > poor environments), even though in the specific context of the visual search period of our task, those environmental objects had no further value as reward predictors and had no instrumental value for obtaining reward. This increased attention or bias to environmental fractals resembles value-driven salience. The effect that association of objects with reward increases gaze at irrelevant stimuli previously associated with reward has been found in a range of primate and human studies [54–57], and may play a crucial role in driving the extended exploration of environments associated with greater reward probability.

These processes could be sub served by the functions of a recently uncovered basal ganglia circuit, including the caudate tail and caudal dorsolateral substantia nigra, which promotes gaze shifts to objects based on a stable, context-independent estimate of their long-term statistical associations with reward or task relevance [66]. Thus, in addition to this circuit’s role in directing gaze to specific ‘good’ vs. ‘bad’ objects within an environment [66], it might also help animals navigate *between* foraging environments by shifting their gaze to ‘good’ vs. ‘bad’ environments as a whole.

Finally, we found that the reward-related bias was accentuated during the search task in which rewards were often probabilistic and uncertain. This suggests that the magnitude of the bias could be influenced by a recently uncovered cortex-basal ganglia circuit including the anterior cingulate cortex, dorsal striatum, and anterior and ventral pallidum, that motivates gaze shifts to seek information to reduce uncertainty about future rewards [67–69]. We found some evidence that this was the case. In our task the target’s identity–reward or no reward–provided the key piece of information to resolve uncertainty about the trial’s outcome. Hence, based on previous work [67,70,71], we would expect direct saccades to the target to be enhanced when reward was uncertain. Indeed, we found that initial, direct saccades to the target occurred with higher probability and shorter latency in environments with uncertain rewards, and were especially rapid when the target conveyed information that was surprising and led to a large change in reward expectations (S4 Fig). As expected from previous work, this influence tapered off after the target was found and its information was collected. Hence, this process could participate in wide range of information seeking behaviors to resolve uncertainty or conflict, and to confirm the subject’s beliefs.

Importantly, when considering the total time spent during visual search, the reward-related bias was modestly enhanced by uncertainty but was still clearly present even in environments where the outcome was certain (S1 Fig). An important topic for future work will be to discover how neural circuits that direct gaze to search for rewards and direct gaze to reduce uncertainty cooperate and compete with each other in visual search [69].

To understand how the monkeys’ object inspection strategy could influence their behavior in natural environments, we extended the classic patch-leaving model of foraging to add the naturalistic requirement that objects in the environment must be inspected to reveal their hidden, variable rewards. In this view, the classic marginal value theorem for modeling this behavior provides the agent with perfect knowledge of the gain function indicating the future rewards that each patch will provide in the future; whereas in our object-inspection foraging model the agent must gather this information for itself. Specifically, the marginal value theorem is designed to model decisions about when to switch *between* patches, but does not model decisions made while foraging *within* a patch, such as what objects to inspect, what actions to take, and what rewards to consume. This is why, the theorem must be provided with a summary of the results of the agent’s within-patch decisions as one of its inputs (in the form of the gain function). By contrast, the main purpose of our object-inspection model is to model decisions while foraging *within* a patch, specifically decisions about when to inspect objects vs. consume their rewards. The agent therefore faces an additional stage of decision making: the agent must decide how much time to spend inspecting potential sources of value to discover their hidden rewards, and how much time to spend exploiting this knowledge by gathering and consuming those rewards. Our model is complementary to recent work that extended the marginal value theorem to model saccadic foraging for visual rewards [72]. That work focused on environments that contained one or two objects, and modeled the precise dynamics of gaze as participants “consumed” the visual reward (e.g. velocities of saccades and durations of individual fixations). By contrast, we focus on environments that contain many objects with diverse values, and model the macro-scale dynamics of gaze as animals accumulate knowledge about their outcomes and decide whether they are worth taking action to harvest and consume in the future.

We found that the optimal policy for foraging in the object-inspection model was similar to the reward-related bias we observed in monkey behavior: a policy of inspecting more objects in rich environments, especially when the targets revealed thus far had low value. This type of policy was robust to different formulations of the model, and is consistent with human visual search in environments with varying numbers of targets [73]. This policy makes intuitive sense. In a poor environment few objects are likely to yield rewards worth taking the time to consume. In a rich environment many objects are likely to yield worthwhile rewards, so it is worth inspecting many objects to get a full picture of the buffet of rewards that are available before starting to consume them.

Crucially, monkeys continued to use their strategy over the course of extensive training even though they were never incentivized to exhibit this behavior in our experiments. In fact, by using this strategy they obtained a considerably lower reward rate than if they had simply accepted the target immediately on each trial and moved on with the task. This may suggest that this reward-related bias is an innate or developmentally learned strategy that monkeys employ when they are visually searching for rewards, and that it is potent enough that animals never overcame it even after extensive training. In this sense, this strategy would be similar to other well-documented natural learning and decision strategies that persist even in experimental environments where they appear to be disadvantageous, such as being biased to choose options that have recently given rewards [74] and that give rewards sooner rather than later (temporal discounting) [75]. Our modeling work shows the potential for this strategy to be beneficial in naturalistic foraging scenarios, but further work is needed to uncover the specific algorithm and its neural circuit implementation the brain.

Our task used a “precue” to indicate to the animals the probability of rewarded versus unrewarded targets. The precues could have also made the visual search easier. Future studies must examine the impact of attentional/cognitive load and effort on visual search strategy and persistence. Also, in our study the response time correlations with reward probability were not linear. This may have arisen due to the interactions of effort and beliefs about reward or due to other non-linearity in valuation processes.

A final important topic that ought to be studied by future research will be to assess how environmental reward statistics, and other features of natural environments, mediate search strategies and how these strategies facilitate adaptive foraging behaviors. It has been reported that monkeys achieve a higher reward rate in a naturalistic patch-leaving task than one would predict from their behavior in a standard binary choice task [76]. Our results suggest that monkeys might be able to achieve high reward rates in even more complex environments, as long as the environments are a good match to their foraging strategy–that is, environments with multiple objects that have a wide variety of values, and that must be inspected to reveal their variable, hidden outcomes. If so, this may help to explain why these reward-biased strategies are such ubiquitous and persistent drivers of human and animal behavior, both in the laboratory and outside of it.

## Methods

### Ethics statement

All procedures conformed to the Guide for the Care and Use of Laboratory Animals and were approved by the Washington University Institutional Animal Care and Use Committee.

#### General procedures

Two adult male rhesus monkeys (Macaca mulatta) were used for the behavioral experiments (Monkeys M and B). A plastic head holder was affixed to the skull under general anesthesia and sterile surgical conditions. After the monkeys recovered from surgery, they participated in the behavioral experiments. The task was developed in Matlab (Mathworks, Natick, MA) with the Psychophysics Toolbox extension. Eye movements were monitored using an infrared video camera (Eyelink, SR Research). Juice, as reward, was dispensed using a gravity-feed solenoid delivery reward system (Crist Instruments).

#### Tasks

*Single fractal saccade instrumental conditioning task*. In order to associate certain visual fractal objects with either reward delivery or no-reward delivery, monkeys participated in an instrumental conditioning task. Visual fractal objects served as targets for saccadic eye movements [77]. Each trial began with the appearance of a white trial-start cue (TS) in the center of the screen. Monkeys had to fixate the TS for 0.4 seconds to initiate the trial. Then, the TS disappeared coincident with the presentation of the target fractal in one of 4 radially symmetric locations (12 radial degrees from the center, canted at 40, 130, 220, and 310 degrees). Monkeys had 5 seconds to fixate the target fractal (defined as gaze position remaining within 2.5° of the center of the target fractal) for 0.75 seconds to complete the trial. Half of a second following successful fixation of the target fractal the outcome was delivered (juice reward or no juice) and the target fractal disappeared. Subsequent inter-trial-intervals were 1–2 seconds long. If the monkeys failed to fixate the target for 0.75 seconds within the 5 second limit, the trial was repeated.

Using this procedure, moneys were conditioned to associate 14 fractals with reward and 14 fractals with no rewards. Training was assessed by the emergence of a response time bias. Monkey M displayed slower response times during no reward trials than reward trials after 10 sessions of training. Monkey B displayed slower response times during no reward trials after just 3 sessions. Monkeys continued instrumental conditioning to verify that the results were replicable across sessions. After the replicability and stability of these effects were confirmed, monkeys began training on the probabilistic search task in which some of the same visual fractal target objects served as visual targets.

*Probabilistic visual search task*. Monkeys searched for one of 8 (4 reward and 4 no-reward) visual target objects previously associated with reward or no reward. The target objects were embedded in visual environments comprised of 12 previously unassociated visual fractals. These will be called “environmental fractals”. Five distinct environments were each composed of a set of 12 unique fractals, and were associated with 5 distinct probabilities of containing a target object associated with reward (0%, 25%, 50%, 75%, and 100%). The 12 environmental fractals were arranged in 3 ‘rings’ (inner, middle, and outer) each containing 4 fractals in a radially symmetrical arrangement. Specifically, the inner ring was created by placing 4 fractals each at 6° eccentricity, positioned at radially symmetric locations along the cardinal axes, and then rotating the ring around the center of the screen by -22.5 degrees. The middle and outer rings were created by the same process, with 15° and 17.5° eccentricities and +22.5 degrees and -22.5 degrees rotations, respectively.

A search trial began with the appearance of a green TS in the center of the screen. Monkeys had to fixate the TS for 0.4 seconds to initiate the trial. Then, the TS disappeared coincident with the presentation of a visual environment without the visual target. This *precue* informed the monkey of the probability of reward in the upcoming search. Following 0.5 seconds, the environment disappeared. After a 1–2 second period, a white central fixation spot appeared in the center of the screen that the monkey had to again fixate for 0.4 seconds. Then the fixation spot disappeared, and the environment was presented again with the target fractal embedded in one of four locations (the same four locations as used in the single fractal saccade instrumental task). Monkeys had 5 seconds to fixate the target for 0.75 seconds to obtain the outcome of the trial associated with the target (reward or no reward). After 0.75 seconds of continuous fixation, the environment disappeared, but the target remained on the screen, and the outcome was delivered after an additional 0.5 seconds. The target fractal disappeared coincident with reward delivery, and the 1–2 second inter-trial-interval began. If the monkeys failed to fixate on the central green TS, the trial would repeat from the green TS following a 1–2 second delay. If the monkeys failed to fixate the white fixation spot, or failed to fixate the target for 0.75 seconds, the trial would repeat starting with the white fixation spot following a 1–2 second delay. Following training monkeys displayed a reward-related search initiation bias–a classic measure of motivation often used to determine monkeys’ understanding of cue values (e.g. [67,78,79]. The time to fixate the white fixation spot was correlated with reward probability in both monkeys (p < 0.001; r = -0.089 in Monkey M; r = -0.175 in Monkey B).

During the visual search task, ~25% of the trials were single fractal trials. The animal could tell whether a trial was going to be a search trial or a single fractal trial based on the color of the first stimulus that appeared on each trial–a green TS for a search trial, or a white TS for a single fractal trial. All target fractals appeared with equal frequency in the single fractal trials. To compare the effects of the environmental precues between search trials vs. single fractal trials, the environment was precued to the animal on a subset of single fractal trials, using the same precue procedure as was normally used during search trials (except that unlike search trials, the environment was only presented during the precue period, not during the search period).

In order to test whether the reaction time bias across environments observed in Search trials carried over to the isolated targets in Single Fractal trials, the monkeys were trained on a second set of 5 environments featuring the remaining 20 target fractals learned in the single fractal instrumental task. However, unlike the first set where the same 8 fractals were used as targets across all of its environments, the second set of environments used a different, unique pool of 4 possible target fractals for each one of its environments (5 environments x 4 unique target fractals per environment = 20 unique target fractals total). These pools remained consistent across sessions, and each fractal in a pool appeared with equal frequency within its environment. To ensure that this was the case, the ratio of reward and no-reward targets in each pool was set accordingly. For instance, the 75% environment pool was composed of 3 reward target fractals and 1 no-reward target fractal. Search durations in the Search task for both environment sets displayed similar modulation by the reward probability of the environment and the value of the target fractal, and so were pooled for most analyses (S1–S5 Figs and S1 Table). After associating each of the new environments with a unique and stable group of target fractals, the Single Fractal reaction times of the target fractals associated with the different environments were evaluated.

In a control experiment to compare the effects of the environmental precues between search trials vs. single fractal trials, the environment was precued on all trials in five sessions (n = 2245 trials in animal B), using the same precue procedure as was normally used during search trials (except that on single fractal trials the environment was only presented during the precue period, not during the search period).

In a control experiment to test whether the effects depended on target fractals being presented at distinct locations from environmental fractals, the target fractal was not presented at one of its usual four locations and instead replaced a random one of the environmental fractals in the outer two rings (n = 3468 trials in animal M). As reported in the text, this produced a very similar pattern of behavior.

#### Data processing and statistics

Search duration was defined as the time from when the white start cue turned off until the monkey began a successful fixation of the target fractal (the fixation period of 0.75 seconds was not included in the search duration). Search duration analysis included repeat trials; similar results were observed if they were excluded.

Discriminability between different distributions of response times was quantified using the area under the receiver operating characteristic (ROC) [80,81]. All correlations used Spearman’s rho (rank correlation) and were evaluated for significance (p < 0.05) using 10,000 permutations unless otherwise noted.

Saccades in all analyses were defined by interpolating between sampled eye positions to regularize the data to 1-millisecond spacing, finding the velocity by taking the derivative, then smoothing the eye velocity using a 15-millisecond wide moving mean, and defining saccades as any period of an eye movement that exceeded a threshold velocity of 0.025°/millisecond. Saccade start and end positions were defined as the gaze locations at the start and end of super-threshold periods. Saccades covering less than 1° of distance were ignored to avoid incorporating saccades made within the visual area of the same fractal object. Direct initial saccades were defined as initial saccades ending within 1.5° of the center of the target fractal. Direct trials where the monkey blinked during the initial foveation of the target were excluded.

In direct trials where the monkey subsequently looked away rather than completing the target fixation, the duration of the initial fixation on the target was determined. Further, the persistent search duration, defined as the time from when the monkey looked away from the target fractal until the end of the search period, was determined. This is a measure of the search duration after the monkey has already identified the target fractal, as indicated by the direct initial saccade, but remained in the environment and explored additional fractals rather than ending the trial by finishing fixation on the target. This suggests that this measure would be minimally affected by perceptual modulations, and instead would primarily reflect search strategy.

The position of the monkeys’ gaze during search (Fig 4A and 4B) not including the terminal fixation was evaluated. The screen was divided into a grid of 100x100 bins in a 61°x61° square centered on the center of the screen. The proportion of time spent in each bin was evaluated for each environment and reward condition, by dividing the number of milliseconds during which gaze was in each bin by the number of trials (Fig 4A).

To further quantify gaze, we defined a gaze window for each object (for each fractal and for the TS), consisting of the nearest 77 bins to the center of that object (approximately a circle with 3° radius). The number of inspected environmental fractals was defined on each trial as the number of environmental fractals for which the gaze was in their window for at least 50 ms. The total search duration was divided into four components: gaze at environmental fractals, gaze at target fractal, gaze at the starting position (the TS window at the center of the screen), and all other search time. The total search duration’s reward-related variance was defined as the variance of a vector of 8 numbers, indicating the mean total search duration for each of the 8 combinations of environmental reward probability and target reward value. The percentage of this variance explained by each component of search was defined as the percentage by which the variance was reduced after subtracting that component of search from the total search duration. Finally, each component’s modulation by reward-related variables was quantified by fitting its single trial durations with an ordinary linear regression model with two regressors, p(reward) (a probability between 0 and 1) and reward (0 for no reward, 1 for reward). We used this simple model with main effects because a version with interactions produced very similar results.

To test the distractibility of the monkey during search, an additional version of the search task was developed. Here, during half of the trials a random member of the middle ring of the environmental fractals was replaced by a novel fractal. The proportion of search spent gazing at the novel fractal was determined by finding the proportion of the search period of each trial that was spent within 1.5° of the novel fractal distractor.

To test how the reward-related behavioral bias influenced the actual reward rate during search, we calculated a ‘reward rate ratio’ (RRR) in specific tasks or task conditions. The RRR measured the degree to which the animal’s actual reward rate differed from the reward rate they would have achieved if their behavior was not influenced by target value, and instead they consistently performed in the manner they did on reward trials. It was defined as RRR = (actual reward rate) / (theoretical reward rate if the animal always behaved as they did on reward trials). We calculated the two terms in this equation as follows. First, to calculate the animal’s actual reward rate during search, we considered all trials in which the animal successfully reached the point during the task when the search array appeared on the screen (Fig 1A and 1B, “Search” period). This included both correct trials (where they successfully completed the search) and search error trials (where they failed to find or hold fixation on the target within the allotted 5 second maximum search duration). We defined the actual reward rate as the actual number of obtained rewards on those trials, divided by the total time spent during those trials between the onset of the search array and the end of each trial. On correct trials, this included the search period, fixation on the target period, outcome period, and inter-trial interval. On search error trials, this included the search period and the inter-trial interval. Taking these terms together, the reward rate was defined as the expected number of rewards per correctly performed trial, divided by the expected post-search-array time per correctly performed trial, expressed as the equation: E[reward | correct] / (E[post-search-array time spent | correct] + E[# search error trials per correct trial]*E[post-search-array time spent | search error]). Second, to calculate the theoretical reward rate if animals always behaved as they did on reward trials, we re-calculated the reward rate after substituting the key behavioral measures from non-rewarded trials with the corresponding behavioral measures from rewarded trials (i.e. the mean correct search duration and the search error rate). To test whether the RRR was different from 1, we calculated the 99.9% bootstrap CI of each RRR and tested whether it excluded 1. To test whether the RRR was different between conditions, we calculated the 99.9% bootstrap CI of the difference between their RRRs and tested whether it excluded 0.

#### Modeling

The object inspection foraging model was defined as follows. The setting contained 5 environments, representing five levels of richness. Upon entering a new patch, the patch was drawn uniformly at random from the set of 5 environments. Each environment contained 9 objects, which were each associated with a distinct probability distribution of reward sizes. The reward probability was an exponentially decaying function of object number, so that the first object had the highest expected reward value and the last object had the lowest expected reward value. Specifically, object *i* in environment *env* had a reward probability of *p_max_*(*env*) × 2^-*τ*(*env*) × (*i*-1)^, where *p_max_* is the reward probability of the first object and *τ* controls how the reward probability decays with object number. Because reward was probabilistic, a given object sometimes delivered reward and sometimes delivered no reward, and the rewards it delivered were sometimes small and sometimes big. Specifically, if an object delivered reward on a given encounter, then the reward size that was revealed upon inspection and made available for consumption was set to a random integer between 1 and 5. If it did not deliver reward, the reward size was set to 0.

To give the 5 environments different levels of richness, we gave their objects different reward distributions. In the analysis shown in Fig 5, we set the environments to have different *p_max_* (0.1, 0.3, 0.5, 0.7, 0.9 for environments 1–5, respectively) and to all have the same *τ* = 0.7. Thus, the object reward probabilities for the different environments were simply scaled versions of each other. We also tested other ways of altering the richness of the environments, including setting the environments to have the same *p_max_* but different *τ*, or setting them to have the same *p_max_* and *τ* but different distributions of reward sizes when reward was delivered. These produced qualitatively similar results (S3 Fig).

The agent’s behavior was divided into inspection and consumption phases. This is based on the intuition that in our task and in many natural environments, a common organization of behavior can be approximated as an information-gathering stage followed by a reward-collecting phase, since visually inspecting objects (e.g. often requiring only eye movements) is generally faster and cheaper than taking action to obtain and consume their rewards (e.g. often requiring body movements and effortful manipulation). During the inspection phase, the agent could choose to inspect the next-best not-yet-inspected object, or to end the inspection phase and begin the consumption phase. Inspecting an object revealed its reward outcome (and incurred a time cost of *t_inspect_* = 0.5 seconds). During the consumption phase, the agent could choose to consume the next-biggest reward (at a time cost of *t_consume_* = 1.25 seconds), or end the consumption phase and travel to a new random patch (at a time cost of *t_travel_* = 8 seconds). These time parameters were set to roughly match the analogous time intervals from our visual search task for visual inspection of an object, holding fixation on a target and acquiring and consuming the reward, and the time interval between the end of one trial and the first moment in the next trial when a response could be initiated. We obtained similar results for a range of settings of these parameters.

To model temporal discounting behavior, rewards in these environments were set to decay over time, which would incentivize the agent to collect rewards sooner rather than later. This is based on previous work in foraging theory, which modeled temporal discounting as the result of environmental constraints such as long time delays coming with greater risk of being interrupted before being able to collect the reward [26,82–84]. Here, to model this in as simple a manner as possible, we set the amount of reward obtained from an act of consumption to be *r* × (1-*k*)*^tdelay^*, where *r* is the revealed reward size, *t_delay_* is the time between the start of the trial and the moment of consumption, and *k* is the temporal discounting term. High *k* implies faster decay of reward value with time. We set *k* = 0.275 to roughly match the amount of temporal discounting in this species in similar laboratory tasks (e.g. a reward losing half of its value after a delay of ~2.2 seconds). We obtained similar results for a range of settings of this parameter, as long as it was not too close to 0 or 1. The effect of temporal discounting was to incentivize a shift from inspection to consumption in situations where a large amount of reward had already been revealed. That is, after many rewards had been revealed the effective cost of time became higher, since spending *t_inspect_* to inspect an additional object would be delaying the consumption of a larger amount of reward.

We defined the optimal policy π* as the policy for making inspection and consumption decisions that maximized the reward rate (i.e. the reward per second). As stated by the marginal value theorem [35], the optimal policy for consumption decisions depends heavily on knowing the global reward rate that it expects to obtain (averaged over all environments), which we will refer to as *R_global_(*π*). However, note that *R_global_(*π*) is itself a function of the current policy. Thus, to compute the optimal policy we used an iterative procedure. We started by initializing *R_global_(*π^0^*)* = 0. We used the dynamic programming procedure described below to compute the optimal policy, π^1^, given the assumption that the global reward rate was *R_global_(*π^0^*)*. We then calculated the true global reward rate under that policy, *R_global_(*π^1^*)*, and iteratively repeated this process. This produced a sequence of policies π^2^, π^3^, …, π*^Niter^* and corresponding global reward rates *R_global_(*π^2^*), R_global_(*π^3^*), …, R_global_(*π*^Niter^)*. We found that after *N_iter_* = 40 iterations this process reliably converged to a stable policy and reward rate, which we then designated as the optimal policy π* and its reward rate *R_global_(*π*).

To compute the optimal policy given *R_global_*, we used dynamic programming. We defined the state*s* of the environment as all possible combinations of (a) the environment identity, *env*, (b) the number of objects inspected so far, *N_inspected_*, (c) a vector containing a sorted list of the revealed reward sizes of those objects, *r*. This yielded a total of 25025 states. Each state had two possible actions *a*: *inspect* the next best object, or *consume* the revealed rewards and travel to a new patch. The value of a state-action pair V(*s*,*a*) was computed using the equations below, as the expected reward rate obtained during a time interval starting at the beginning of a trial in which that state was visited and that action was taken, and lasting for *T_max_* seconds after the beginning of that trial (where *T_max_* = 23.75 s is set to be the maximum possible length of a single trial). The policy in that state π(*s*) was then defined to be *inspect* if V(*s*,*inspect*) > V(*s*,*consume*), and *consume* otherwise. Finally, the value of the state as a whole, V(*s*), was defined as the value of being in that state and following its policy, i.e. V(*s*, π(*s*)).

From a given state, the value of inspecting the next object was simply computed as the expected value of the next state resulting from inspecting that object, based on the probabilities of transitioning from the current state *s* to each possible next state *s’*:

$$V(s,inspect)=\sum_{s’}p(s’|s)\times V(s’)$$

The value of consumption was computed based on the time-discounted reward that could be gained by consuming the *i* biggest revealed rewards (each of which was appropriately discounted based on the elapsed time thus far during the trial), traveling to a new patch, and thereafter obtaining the average global reward rate for the remainder of the total time interval being considered. The value of consumption was set to be the maximum value this could take, optimizing over all possible choices of the *i* best rewards to consume.


V(s,consume)=maxi(((∑j=1:irj×(1‐k)^(Ninspected×tinspect+j×tconsume))+Rglobal×(Tmax‐(Ninspected×tinspect+i×tconsume+Ttravel)))/Tmax)

Note that because the values in this model are reward rates, V(*s,consume*) is in units of reward/s. We first calculated V(*s,consume*) for all states, and then used dynamic programming to calculate V(*s*,*inspect*), π(*s*), and V(*s*) for all states.

To plot the optimal policy’s decision rule, for each setting of *env* and *N_inspected_*, we found the value of consumption for which the policy always chose to consume if V(*s*,*consume*) was higher than that value, and always chose to inspect if V(*s*,*consume*) was lower than that value. This threshold-based decision rule accurately described the optimal policy’s decisions in all states.

To compute the optimal policy under the constraint that the agent only had access to a subset of the state variables, we augmented the dynamic programming computations using a partially observable Markov decision process (POMDP) model which estimated the probability distribution over the missing pieces of the state using Bayesian inference, and used this state distribution to calculate the values. For example, the model that only had access to *r* and *N_inspected_* could not simply calculate p(*s’* | *s*) using the known statistics of the current environment *env*, because the *env* part of the state was hidden. Instead, it had to calculate p(*s’* | *s, env = env_i_*) separately for each possible environment *env_i_*, use Bayesian inference to calculate the probability distribution over the current environment variable p(*env*), and use this to calculate p(*s’* | *s*) by marginalizing over the unknown environment variable.

## Supporting information

S1 Fig Quantifying the effect of each animal’s reward-related bias on their achieved reward rate. **(A)** Comparison of animal B’s reward rates in the Single Fractal task vs. the Search Task. *Left*: the first key component of the reward rate: the probability of failing to complete the search, on reward vs. no reward trials (red vs. blue). If the animal did not complete the search within the 5 second search period, they had to repeat the trial until they did so successfully. *Middle*: the second key component of the reward rate: the mean duration on successfully completed trials between the onset of the search array and the end of the trial (including both the search duration, the hold period on the target, the time spent receiving the reward, and the inter-trial interval). *Right*: the resulting reward rate ratios (RRRs): the ratio of the reward rate (in rewards per second) that the animal actually achieved, relative to the theoretical reward rate they would have received if they always behaved in the manner they did on rewarded trials (i.e. if they had no reward-related bias in behavior between reward vs. no reward trials). *** indicates that the Search Task has a lower RRR than the Single Fractal task, indicated by the 99.9% bootstrap CI of their difference excluding 0. *Right*: *** indicates that uncertain trials have a lower RRR than the certain trials, indicated by the 99.9% bootstrap CI of their difference excluding 0. **(B)** Same as A, but comparing Search Task certain trials (0% and 100% reward) vs. uncertain trials (25, 50, and 75% reward). Both certain and uncertain environments were associated with the same average reward probability when taken as a whole (50% reward). However, we reasoned that uncertain environments could produce a stronger net reward-related bias due to the greater contrast between their possible target values. For example, a reward may be more motivating when it is better than the expected outcome (e.g., in the 50% reward environment) than when the same reward is fully expected (in the 100% reward environment), consistent with the search durations we observed in our task (Fig 2C). Indeed, this was the case: the reward-related behavioral bias produced a greater reduction in reward rate in uncertain reward environments. **(C,D)** Same as A,B, for animal M.
(TIF)

S2 Fig Object inspection behavior was consistent in both animals. Crucially, the number of inspected objects, and the total search duration, were negatively related to target reward value (red < blue) and positively related to environmental reward probability (red and blue lines both have positive slopes). Furthermore, the component of search duration that was predominantly responsible for these effects was gaze at the environmental fractals. **(A-E)** Data from animal B. (A-D) are the same format as Fig 4B–4E. (E) shows the mean and SD of each of the four components of search duration. Error bars are ±1 SE. For this animal, the component of search with the longest mean duration and greatest variability was the component during which gaze was on the environmental fractals. **(F-J)** Same as A-E, for animal M. For this animal, the component with the longest duration was when the gaze was on the initial fixation location, while the component with the greatest variability was when gaze was on the target (note that this only includes time when the gaze was on the target and then the animal looked away, i.e. *before* the animal began the final, required hold duration to successfully complete fixation on the target to complete the trial).
(TIF)

S3 Fig The foraging model’s results are robust to different ways that environments can be rich vs. poor, as long as they contain objects with hidden, variable rewards. **(A)** Summary of results for the model shown in Fig 5, that sets environmental richness using the *p*_*max*_ parameter. *Left*: object reward probabilities (p(reward), colored lines for each of the five environments), and the probability distribution of reward sizes for when reward is delivered (p(reward size | reward), black line, same for all five environments). Environments have different reward probabilities of the best object (*p*_*max*_); they decay with object number at the same rate (τ); and all reward sizes are uniformly distributed between 1 and 5. *Middle*: reward rates achieved for the four policies which have access to different state variables; Policy 4, which has access to all of the state variables, produces the optimal reward rate. *Right*: object inspection behavior of Policy 4 in each environment on trials when the objects have outcomes similar to the Search Task’s no reward trials (no objects give reward) or reward trials (the first inspected object gives a reward of size 3 and all other objects give no reward). As in the behavior of actual animals, the number of inspected objects is negatively related to object reward value (red < blue) and positively related to the environment’s richness (red and blue lines both have positive slopes). **(B)** Alternate formulation that sets environmental richness using the τ parameter. *Left*: object reward probabilities in all environments start at the same *p*_*max*_, but decay with object number at different rates. *Middle*, *Right*: Policy 4 still has an advantage over all other polices, and produces qualitatively similar object inspection behavior (red < blue, red and blue lines both have positive slopes). **(C)** Alternate formulation that sets environmental richness using the reward size distributions (*Left*, colored lines, rich environments more likely to give big sized rewards), while fixing the reward probabilities in all environments to be the same exponentially decaying function of object number (Model C1, top row) or to be a fixed constant (Model C2, bottom row). *Middle*, *Right*: Policy 4 still has the highest reward rate, and produces similar object inspection behavior (red < blue, red and blue lines have positive slopes). **(D)** Alternate formulation that sets environmental richness using a combination of all three parameter settings from models A, B, and C1 (*Left*, though only varying each variable by 1/3 of its original extent, in order to roughly match their net effect on the environments). This is to test whether these three variables, which are *all* likely to be relevant in rich vs. poor natural environments, have similar effects on the optimal policy when they all occur at once. *Middle*, *Right*: Policy 4 has a similar (or if anything larger) advantage over the other policies, and produces similar object inspection behavior. **(E)** Null model in which environments and objects do not vary in richness: all objects have the same reward probability and reward size (Left). *Middle*, *Right*: Policy 4 no longer has an advantage over the simpler Policy 3 which ignores the environment’s identity, and instead shows simple ‘all or nothing’ object inspection behavior (red < blue, red and blue lines have flat slopes).
(TIF)

S4 Fig Direct saccade properties as a function of uncertainty and its resolution. We found two pieces of evidence that direct saccades were influenced by environments with uncertain vs. certain rewards. First, averaging over all targets, animals tended to have the highest probability of making direct saccades, and to do so with the fastest response times, in an environment that had intermediate reward probabilities and hence had uncertain rewards (A). Second, animals tended to make direct saccades with faster response times when the information conveyed by the target was more unexpected or surprising: response times to reward targets were fastest in poor environments, while response times to no reward targets were fastest in rich environments (B). This resembles a well-known gaze bias linked to Bayesian inference, in which individuals can be faster to detect and respond to new information that is unexpected and can lead to a larger update in the individual’s beliefs (Bayesian surprise [85]). **(A)** The probability (top) and response time (bottom) of direct saccades from each animal (columns), as a function of the environmental reward probability. This panel pools both reward target and no reward target trials. Error bars are ±1 SE. Black text indicates correlation and its p-value (permutation test with 10,000 permutations). As the environmental reward probability increased, both animals had significantly higher direct saccade probabilities (positive correlations) and faster response times (negative correlations). However, behavior was not simply a monotonic function of reward probability, and was consistent with there being a component related to uncertainty. Gray text indicates that the highest direct saccade probability and fastest response times were *not* in the environment where reward was 100% certain, but rather, in an environment that had a high reward probability but some degree of uncertainty (75%). This was present as a significant effect and a trend in animal B, and highly significant effects in animal M (p-values indicated by gray text). **(B)** Analogous plot separately for reward and no reward trials (red and blue). The direct saccade probability (top) was modulated by environmental reward probability similarly for both targets, i.e. in a positive direction (positive correlations). By contrast, direct saccade response times for the two targets were modulated oppositely–saccades to the reward target were generally fastest at low reward probability (positive correlations, significant in both animals), while saccades to the no reward target were generally fastest at the high reward probability (negative correlation significant in animal M; no significant correlation in animal B, but strongly and significantly faster at the highest reward probability than all other reward probabilities, rank-sum tests, all p < 10^−14^).
(TIF)

S5 Fig Exploratory gaze distribution is visible even in single-fractal trials. Under single fractal conditions, a monkey tends to saccade directly to the target fractal, but may still look around the environment or off the screen. (**A**) In reward conditions the monkey is less likely to look away from the target fractal, and when it does so it is predominantly in a preferred direction off the screen. (**B**) In non-reward conditions the monkey is more likely to look away from the target, and while the trend of looking to the right of the screen is retained, gaze is also directed at blank space on the screen.
(TIF)

S1 Table Key results separately for each animal and condition. The table shows key statistics from each individual animal as well as pooled analysis of both animals. These include statistics of (1) search durations for each task and for Reward (red) and No Reward (blue) conditions, as well as the ROC area for discriminating Reward from No Reward conditions and how that ROC area differs across tasks, (2) search durations in the Search Task additionally split by environmental reward probability, and reporting the correlation between search duration and reward probability, (3) the same, restricted to the first of the two sets of environmental fractals, (4) the same, restricted to the second set of environmental fractals, (5) analogous statistics for the remaining search durations after animals made direct saccades to the target fractal and then saccaded away from it, (6) analogous statistics for probability of search errors (i.e. failing to complete the Search Task due to not successfully fixating the target fractal within the allotted search time).
(TIF)
