# Supplementary figures and images for: How the value of the environment controls persistence in visual search
Source: PLoS Comput Biol. 2021 Dec 14;17(12):e1009662. doi: 10.1371/journal.pcbi.1009662 (PMC8714092; doi:10.1371/journal.pcbi.1009662)

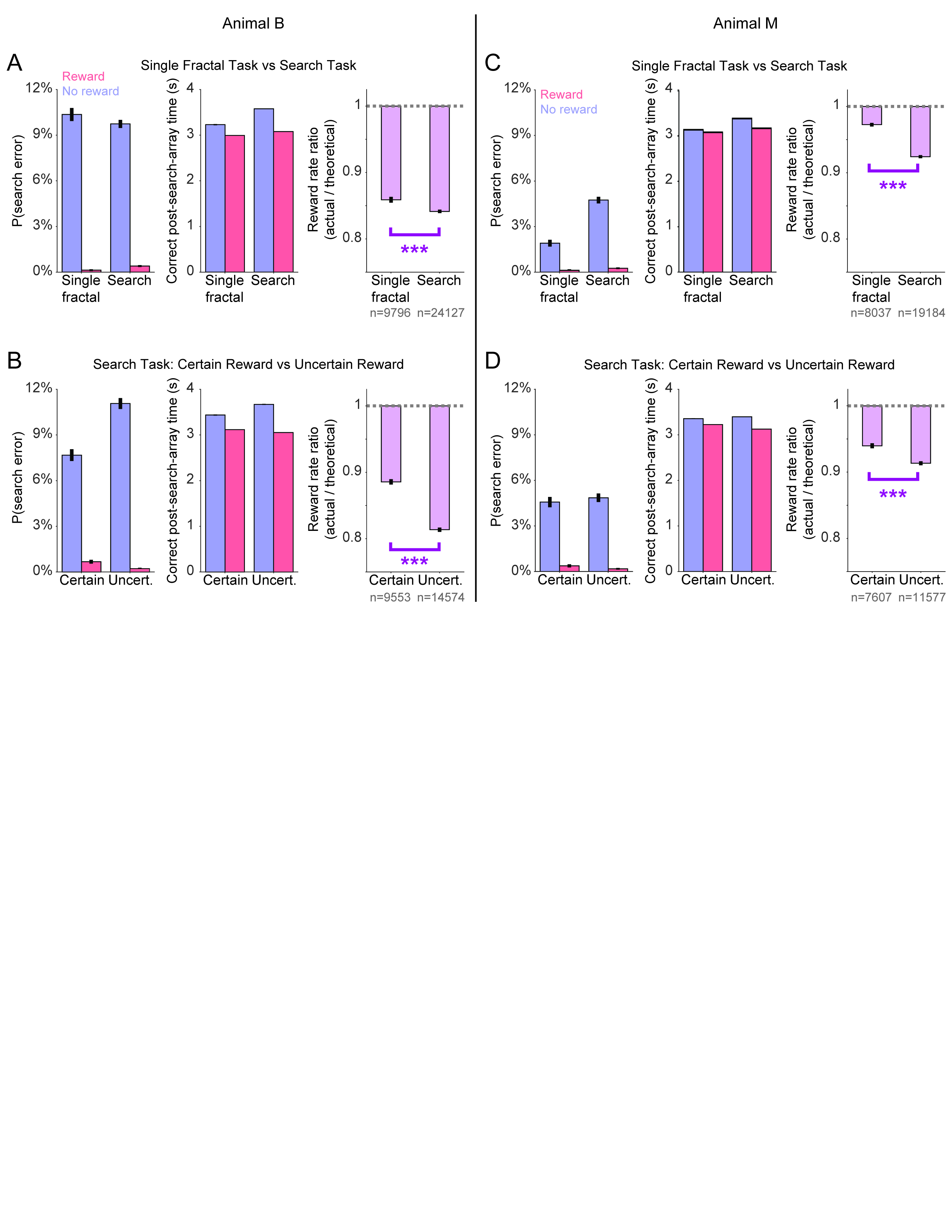

Supplement: S1 Fig — (A) Comparison of animal B’s reward rates in the Single Fractal task vs. the Search Task. Left: the first key component of the reward rate: the probability of failing to complete the search, on reward vs. no reward trials (red vs. blue). If the animal did not complete the search within the 5 second search period, they had to repeat the trial until they did so successfully. Middle: the second key component of the reward rate: the mean duration on successfully completed trials between the onset of the search array and the end of the trial (including both the search duration, the hold period on the target, the time spent receiving the reward, and the inter-trial interval). Right: the resulting reward rate ratios (RRRs): the ratio of the reward rate (in rewards per second) that the animal actually achieved, relative to the theoretical reward rate they would have received if they always behaved in the manner they did on rewarded trials (i.e. if they had no reward-related bias in behavior between reward vs. no reward trials). *** indicates that the Search Task has a lower RRR than the Single Fractal task, indicated by the 99.9% bootstrap CI of their difference excluding 0. Right: *** indicates that uncertain trials have a lower RRR than the certain trials, indicated by the 99.9% bootstrap CI of their difference excluding 0. (B) Same as A, but comparing Search Task certain trials (0% and 100% reward) vs. uncertain trials (25, 50, and 75% reward). Both certain and uncertain environments were associated with the same average reward probability when taken as a whole (50% reward). However, we reasoned that uncertain environments could produce a stronger net reward-related bias due to the greater contrast between their possible target values. For example, a reward may be more motivating when it is better than the expected outcome (e.g., in the 50% reward environment) than when the same reward is fully expected (in the 100% reward environment), consistent with the search duratio [file pcbi.1009662.s001.tif]

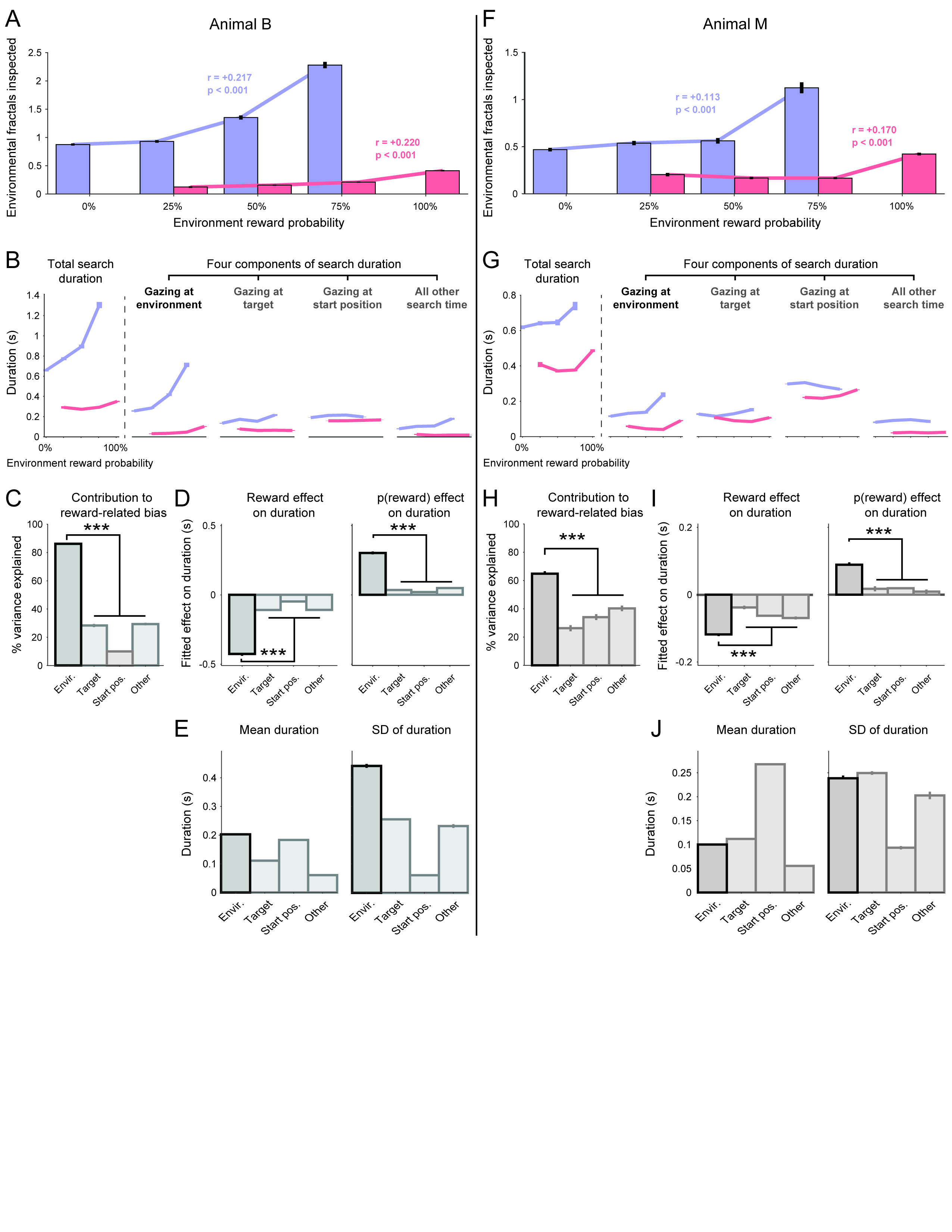

Supplement: S2 Fig — Crucially, the number of inspected objects, and the total search duration, were negatively related to target reward value (red < blue) and positively related to environmental reward probability (red and blue lines both have positive slopes). Furthermore, the component of search duration that was predominantly responsible for these effects was gaze at the environmental fractals. (A-E) Data from animal B. (A-D) are the same format as Fig 4B–4E. (E) shows the mean and SD of each of the four components of search duration. Error bars are ±1 SE. For this animal, the component of search with the longest mean duration and greatest variability was the component during which gaze was on the environmental fractals. (F-J) Same as A-E, for animal M. For this animal, the component with the longest duration was when the gaze was on the initial fixation location, while the component with the greatest variability was when gaze was on the target (note that this only includes time when the gaze was on the target and then the animal looked away, i.e. before the animal began the final, required hold duration to successfully complete fixation on the target to complete the trial). (TIF) [file pcbi.1009662.s002.tif]

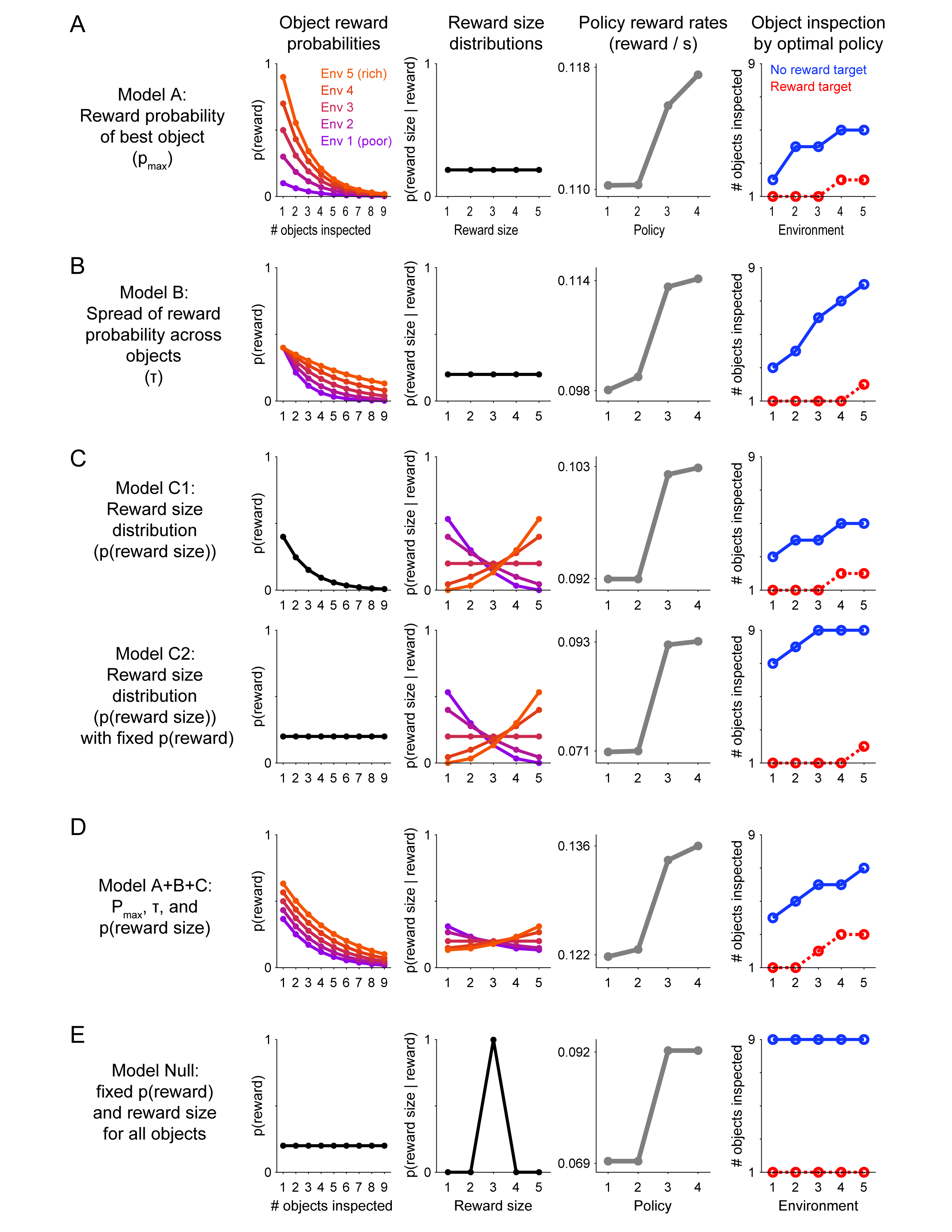

Supplement: S3 Fig — (A) Summary of results for the model shown in Fig 5, that sets environmental richness using the pmax parameter. Left: object reward probabilities (p(reward), colored lines for each of the five environments), and the probability distribution of reward sizes for when reward is delivered (p(reward size | reward), black line, same for all five environments). Environments have different reward probabilities of the best object (pmax); they decay with object number at the same rate (τ); and all reward sizes are uniformly distributed between 1 and 5. Middle: reward rates achieved for the four policies which have access to different state variables; Policy 4, which has access to all of the state variables, produces the optimal reward rate. Right: object inspection behavior of Policy 4 in each environment on trials when the objects have outcomes similar to the Search Task’s no reward trials (no objects give reward) or reward trials (the first inspected object gives a reward of size 3 and all other objects give no reward). As in the behavior of actual animals, the number of inspected objects is negatively related to object reward value (red < blue) and positively related to the environment’s richness (red and blue lines both have positive slopes). (B) Alternate formulation that sets environmental richness using the τ parameter. Left: object reward probabilities in all environments start at the same pmax, but decay with object number at different rates. Middle, Right: Policy 4 still has an advantage over all other polices, and produces qualitatively similar object inspection behavior (red < blue, red and blue lines both have positive slopes). (C) Alternate formulation that sets environmental richness using the reward size distributions (Left, colored lines, rich environments more likely to give big sized rewards), while fixing the reward probabilities in all environments to be the same exponentially decaying function of object number (Model C1, top row) or to be a fixed constan [file pcbi.1009662.s003.tif]

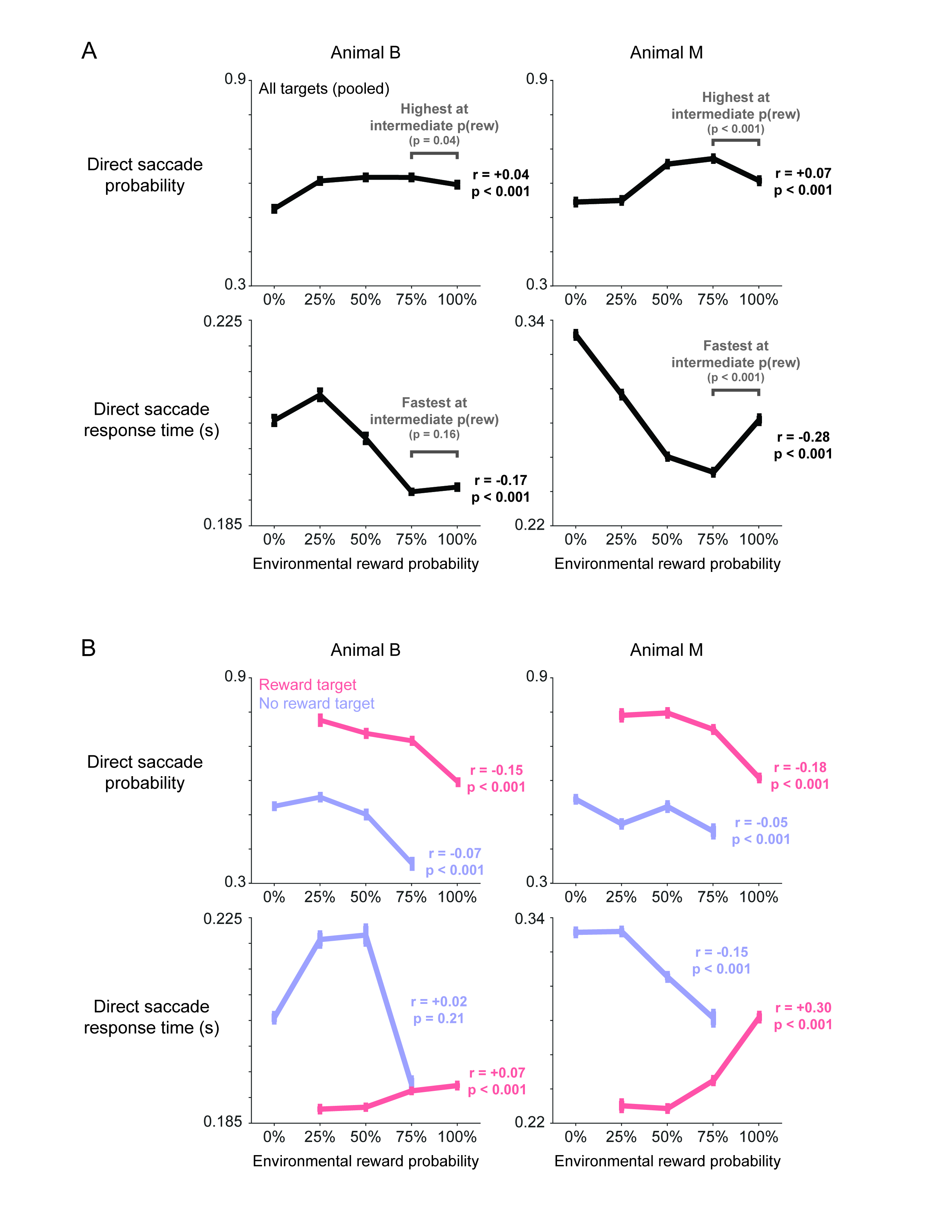

Supplement: S4 Fig — We found two pieces of evidence that direct saccades were influenced by environments with uncertain vs. certain rewards. First, averaging over all targets, animals tended to have the highest probability of making direct saccades, and to do so with the fastest response times, in an environment that had intermediate reward probabilities and hence had uncertain rewards (A). Second, animals tended to make direct saccades with faster response times when the information conveyed by the target was more unexpected or surprising: response times to reward targets were fastest in poor environments, while response times to no reward targets were fastest in rich environments (B). This resembles a well-known gaze bias linked to Bayesian inference, in which individuals can be faster to detect and respond to new information that is unexpected and can lead to a larger update in the individual’s beliefs (Bayesian surprise [85]). (A) The probability (top) and response time (bottom) of direct saccades from each animal (columns), as a function of the environmental reward probability. This panel pools both reward target and no reward target trials. Error bars are ±1 SE. Black text indicates correlation and its p-value (permutation test with 10,000 permutations). As the environmental reward probability increased, both animals had significantly higher direct saccade probabilities (positive correlations) and faster response times (negative correlations). However, behavior was not simply a monotonic function of reward probability, and was consistent with there being a component related to uncertainty. Gray text indicates that the highest direct saccade probability and fastest response times were not in the environment where reward was 100% certain, but rather, in an environment that had a high reward probability but some degree of uncertainty (75%). This was present as a significant effect and a trend in animal B, and highly significant effects in animal M (p-values indicated by gray text). [file pcbi.1009662.s004.tif]

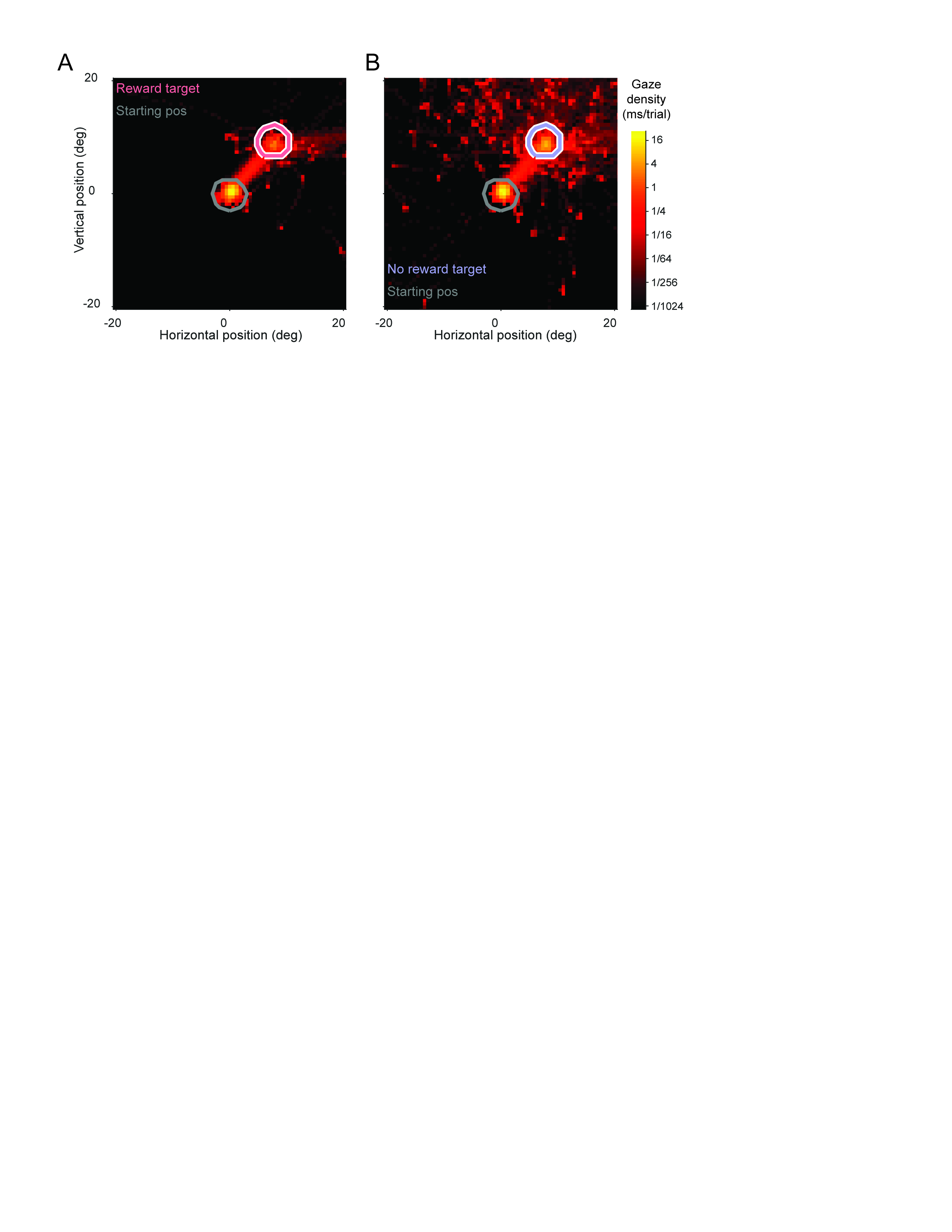

Supplement: S5 Fig — Under single fractal conditions, a monkey tends to saccade directly to the target fractal, but may still look around the environment or off the screen. (A) In reward conditions the monkey is less likely to look away from the target fractal, and when it does so it is predominantly in a preferred direction off the screen. (B) In non-reward conditions the monkey is more likely to look away from the target, and while the trend of looking to the right of the screen is retained, gaze is also directed at blank space on the screen. (TIF) [file pcbi.1009662.s005.tif]
